# Protecting Proteins from Desiccation Stress Using Molecular Glasses and Gels

**DOI:** 10.1021/acs.chemrev.3c00752

**Published:** 2024-04-18

**Authors:** Gil I. Olgenblum, Brent O. Hutcheson, Gary J. Pielak, Daniel Harries

**Affiliations:** § Institute of Chemistry, Fritz Haber Research Center, and The Harvey M. Krueger Family Center for Nanoscience & Nanotechnology, The Hebrew University, Jerusalem 9190401, Israel; † Department of Chemistry, University of North Carolina at Chapel Hill (UNC-CH), Chapel Hill, North Carolina 27599, United States; ‡ Department of Chemistry, Department of Biochemistry & Biophysics, Integrated Program for Biological & Genome Sciences, Lineberger Comprehensive Cancer Center, University of North Carolina at Chapel Hill, Chapel Hill, North Carolina 27599, United States; Lineberger Cancer Center, University of North Carolina at Chapel Hill (UNC-CH), Chapel Hill, North Carolina 27599, United States; Integrative Program for Biological and Genome Sciences, University of North Carolina at Chapel Hill (UNC-CH), Chapel Hill, North Carolina 27599, United States

## Abstract

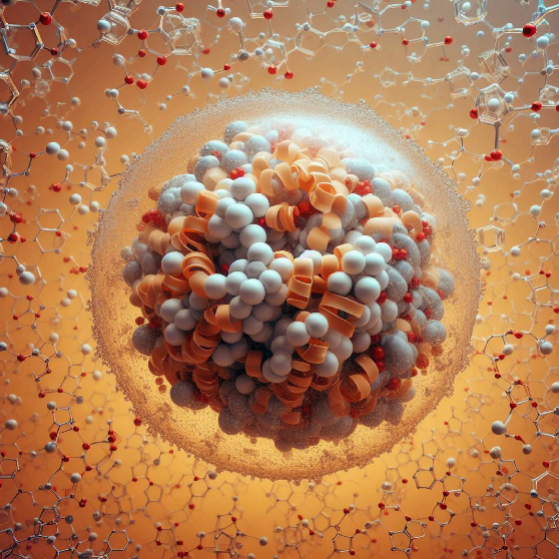

Faced with desiccation stress, many organisms deploy strategies to maintain the integrity of their cellular components. Amorphous glassy media composed of small molecular solutes or protein gels present general strategies for protecting against drying. We review these strategies and the proposed molecular mechanisms to explain protein protection in a vitreous matrix under conditions of low hydration. We also describe efforts to exploit similar strategies in technological applications for protecting proteins in dry or highly desiccated states. Finally, we outline open questions and possibilities for future explorations.

## 1 INTRODUCTION

### 1.1 Protein Stability and Desiccation Stress

Globular proteins are delicate. In solution they exist in a tight but facile ensemble of conformations centered around their native, biologically active state. Many fold via a two-state mechanism, with folding free energies in solution at room temperature and neutral pH of −5 to −15 kcal/mol, an energy comparable to that of a single hydrogen bond.^1^,^2^ Concomitantly, their melting temperatures — the temperature at which half the protein molecules unfold — range from ∼25 to ∼110 °C.^3,4^ This marginal stability has been selected via evolution to allow proteins to maintain the flexibility required for function while preserving their 3D structure under physiological conditions.

The sensitivity of proteins makes them vulnerable to changes in their surroundings. Living creatures must accommodate a variety of stresses brought on by deleterious high or low temperature,^5−9^ salinity,^10^ acidity or alkalinity,^11−13^ radiation,^14^ oxidation,^15^ desiccation,^16^ and even hydrostatic pressure (for deep ocean dwellers).^1,17−20^ Such stresses drive proteins toward or away from their native state, potentially impairing their biological function. Consequently, numerous strategies have evolved to combat these insults and enable proteins (and other biomacromolecules) structure to survive environmental changes.

We focus on desiccation stress. Proteins evolved in water, and their properties change when subjected to desiccation. In the dry state, globular proteins still exist in an ensemble of states, but changes among conformations are no longer facile,^21^ and the conformational ensemble, relative to solution, is “frozen”, or “stuck”. Moreover, removing water is expected to be, and usually is, destabilizing.

The fraction of proteins existing in non-native states under desiccation increases for several reasons. First, removing water negates the hydrophobic effect, the driving force keeping nonpolar side chains buried in the protein interior.^22−24^ Specifically, the molecular underpinning of the hydrophobic effect — the increase in water entropy upon burial of the nonpolar moieties — is moderated with the onset of desiccation. Second, removing the distinction between inside and outside exposes more backbone, which increases the likelihood of non-native hydrogen bonding. The third reason involves hydration of charge. In solution, ionic interactions within and between proteins are weak because water has a dielectric constant that is about 80 times larger than air. Eliminating water increases the strength of native and non-native ionic interactions, both inter- and intra-protein, shifting the delicate balance that holds proteins in their functional form.

Cells are crowded with proteins, with typical concentrations of 100 to 300 g/L.^25−28^ This range is likely the maximal concentration compatible with cellular function. Different proteins exhibit distinct tolerances before they are destabilized due to dehydration and subsequent increase in inter-protein interactions. Ubiquitin, a highly miscible protein, can be concentrated up to 100 g/L,^29^ whereas ribonuclease has a maximum solubility of only 2 g/L.^30^ As a rough estimate of the minimal average hydration of the solvated proteins, consider a radius of gyration of approximately 1.2 nm for ubiquitin^31^ and 1.4 nm for ribonuclease,^32^ and a water molecule with a diameter of about 0.3 nm. Then, the solubility of these proteins translates to an average spatial separation of ca. 13 water layers for ubiquitin pairs and 64 layers for ribonuclease pairs at their solubility limits.^33^ This simple estimate extends, for example, to DNA in plant nuclei, which also reaches concentrations ≈100 g/L,^34^ indicating a comparable minimal hydration level.

**Figure 1:**
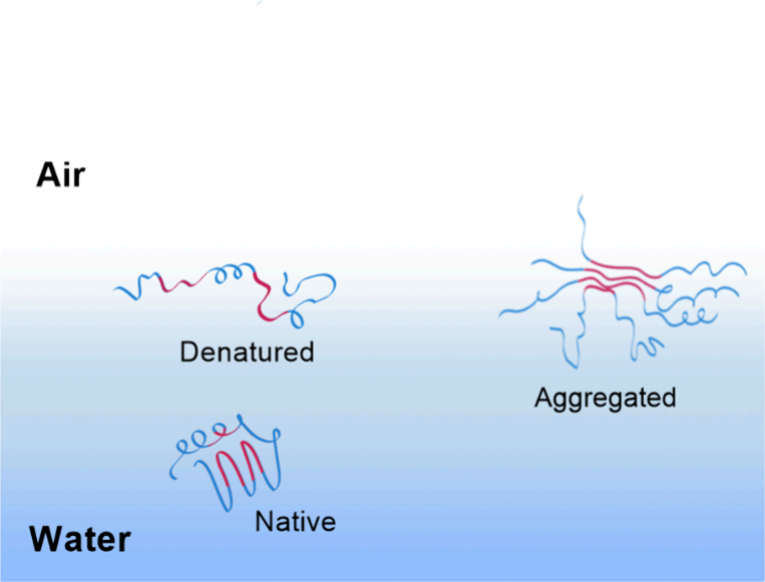
Schematic of proteins in water and at the water–air interface. Proteins tend to denature at the air–water interface, where they expose hydrophobic residues (in red) that are mostly buried in the compact native state. Aggregation may follow as neighboring proteins adhere nonspecifically to each other.

Under dehydrating conditions, water molecules evaporate, and solute concentrations increase, challenging protein stability.^35,36^ The contact of proteins with the air–water interface also increases. Proteins tend to adsorb and denature at the interface, to an extent comparable to their accumulation at water–oil interfaces (Figure 1).^37−39^ This tendency is a result of favorable interactions of hydrophobic protein moieties with the nonaqueous environment presented by air. In aqueous solution, these hydrophobic parts are mostly buried inside the folded protein interior, but hydrophobic interfaces, such as air, tip the balance toward more favorable interactions of nonpolar protein residues with the environment. These hydrophobic interactions lead to exposure of the previously buried nonpolar moieties and promote protein denaturation.

The elevated protein concentration on desiccation is in and of itself an additional challenge that can lead to protein aggregation. The problem arises because at high enough protein concentrations the probability of proteins sticking to each other grows due to the increased probability of protein–protein encounters.^40,41^ This problem is exacerbated by the exposure of hydrophobic parts of the protein that can interact with each other, leading to entangled aggregates (Figure 1). Aggregation is often irreversible, because the free energies required to disentangle the large aggregates exceed that provided by thermal energy.

The long human history of preserving foods by dehydration teaches us that dry proteins may become more susceptible to certain damaging chemical reactions. The main reason for this chemical sensitivity that can lead to irreversible protein unfolding is that, in the denatured state, proteins are vulnerable to interactions with harmful chemical agents. Processes leading to inactivation include oxidation, disulfide interchange catalyzed by reduction, β-elimination, and racemization.^42^ Irrespective of the reason or destabilizing factor, protein unfolding and aggregation are detrimental to the proper function of living organisms. Consequently, biology has evolved mechanisms to counter the negative effects of desiccation.

### 1.2 Biology’s Efforts to Overcome Desiccation Stress

Adaptation allows a wide range of organisms to counteract desiccation stress. Notable among these mechanisms is anhydrobiosis, defined as the ability to lose almost all cellular water and enter a state where metabolism stops but starts again upon rehydration.^35^ Anhydrobiosis allows organisms to avoid the damaging effects of desiccation on proteins, lipid membranes, and nucleic acids. Among the life forms capable of anhydrobiosis are brine shrimp, *Aedes* mosquitoes, and tardigrades. Brine shrimp are marketed to children as “The World’s only Instant Pets” because the dried organisms can be sent by mail and “brought to life” by simply adding water.^43^ Eggs of *Aedes* mosquitoes, associated with viral infections,^44^ can withstand 21-days of desiccation, readily hatching upon rehydration.^45,46^ Tardigrades, popularly known as water bears, are microscopic animals found throughout the world, various species of which are not only capable of anhydrobiosis^46^ but also, among other stresses capable of surviving high amounts ionizing radiation, pressures higher than those found in the deepest ocean trenches, and even the rigors of outer space.^47^ This adaptation allows them to virtually halt their metabolism and growth in dry or suboptimal conditions and later resume normal function when conditions improve by readsorbing water.

In the plant and fungi realm, desiccation tolerance is primarily, though not exclusively, observed in seeds, pollen, and spores. The ability of these reproductive elements to undergo reversible anhydrobiosis facilitates their efficient distribution, even under challenging environmental conditions, and their reactivation only when and where conditions are favorable.^36^

Our understanding of anhydrobiosis is incomplete but we know that cells respond to desiccation, as well as stresses such as freezing, in a variety of carefully regulated ways. One response is to accumulate or synthesize large amounts of additives.^36^ These additives or “protectants” include specialized proteins and small organic molecules, most often sugars and sugar alcohols. During dehydration, solute concentrations grow, eventually reaching the solubility limit of the additive solutes. If dehydrated fast enough, solutions may enter a metastable state and do not crystallize, but instead solidifies into an amorphous glass which shows molecular disorder but retains a solid-like rigidity (Section 2.1). Thus, under extreme desiccation and in the presence of these additives, a vitrified glassy or aerogel-like matrix forms. This matrix stabilizes the embedded proteins, allowing them to maintain their native structure, or preserving their ability to regain native structure, even as the cellular matrix loses nearly all its water.^48^ The vitrified matrix also combats chemical hazards by reducing diffusion of harmful reagents, including oxidative species.^49^ Upon rehydration, and in some cases even in the dry state, proteins remain active.^50,51^

The most prominent sugar accumulated in many organisms is trehalose,^52^ but many others are known (Table 1). In addition, many cells upregulate distinct proteins. Prominent classes are the late embryogenesis abundant (LEA), and cytosolically abundant heat soluble (CAHS) proteins.^27,53,54^ LEA proteins, first detected in plant seeds, have homologues in microorganisms and animals.^35,55^ Typically small hydrophilic and glycine-rich, LEA proteins are disordered when hydrated, but tend to gain structure when dried.^56^ CAHS proteins,^57^ a family found exclusively in tardigrades, are both sufficient and necessary for survival when dried.^58^ More specifically, they support desiccation survival not only in tardigrades but also when expressed recombinantly in yeast and bacteria.^58,59^ Unlike LEA proteins, CAHS protein form strong, reversible hydrogels and aerogels of known secondary structure.^60−63^

**Table 1:** Common Additives in Anhydrobiosis

**System**	**Additive**	**Refs**
bacteria	trehalose	(69)
yeast and fungi	trehalose and glycerol	(16,70)
plants	sucrose, octulose in *Craterostigma plantagineum*, trehalose in *Myrothamnus flabellifolia*, raffinose in *Xerophyta villosa*, galactinol in *X. viscosa,* LEA proteins in most plants	(71−73)
lichen	lichens that establish symbiosis with cyanobacteria accumulate sugars (glucose), but those containing green algae preferentially accumulate polyols (ribitol, arabitol, erythritol, sorbitol, etc.)	(72,74−76)
seeds	sucrose, trehalose, nonreducing oligosaccharides of the raffinose group, LEA proteins	(49,71,77,78)
insects	glycerol, trehalose, LEA proteins	(70,79,80)
tardigrades	CAHS and LEA proteins, trehalose	(81)
brine shrimp (*Artemia*)	glycerol, trehalose, LEA proteins	(82)
nematodes	trehalose	(82−85)

The details of how LEA and CAHS proteins overcome desiccation stress are unclear, but their abundance and tendency toward disorder suggest that they work by actively participating in forming an aerogel or glassy matrix, possibly together with sugars that often accumulate simultaneously. Studies of LEA and CAHS proteins in vitro show that they typically protect other proteins and lipid membranes similarly (or even better) compared to protection that sugars provide, suggesting they act by similar mechanisms.^56,58,64−68^

Although it may seem intuitive that immobilization in a dehydrated matrix stabilizes proteins, the molecular mechanisms are unresolved. Below, we describe some of the observations (Section 3) and the most prominent theories (Section 4) to explain stabilization in the additive-rich dry state.

### 1.3 Engineering Efforts to Stabilize Proteins under Desiccation Stress

The importance of protein stabilization in the pharma, cosmetics, and food industries cannot be overstated. Extending the catalytic lifetime of an enzyme, transporting proteins in their dry state, extending the shelf life of food or drugs, and storage around room temperature all demand methods to stabilize desiccated proteins.^86^ Moreover, some proteins must retain activity in nonaqueous environments, at nonphysiological pH or salinity, or when immobilized to a surface, which imposes similar demands on stabilization. As expected, strategies employed by industry are often similar to those that evolved in biology, including embedding the protein in a glassy matrix or aerogel (Table 2).

**Table 2:** Examples of Protein Desiccation in Glassy Matrices and Aerogels

**Matrix composition**	**Matrix type**	**Embedded proteins**	**Refs**
amorphous SiO_2_	sol–gels, sol–gels plus additives	lysozyme, α-lactalbumin, hemoglobin, metmyoglobin, creatine kinase, hexokinase, heme proteins, glucose oxidase, peroxidases, catalase, tyrosinase, lactate dehydrogenase, urease, bovine carbonic anhydrase, alkaline phosphatase, acetylcholinesterase, butyrylcholinesterase, acid phosphatase, xylanase	(87−95,101−103)
amorphous SiO_2_	aerogel	glucose oxidase, acid phosphatase, xylanase	(95)
Au-immobilized protein in SiO_2_	aerogel	cytochrome *c*	(104)
sucrose, trehalose, and leucine	sprayed or lyophilized glass	lysozyme, rubella vaccine, and influenza antigen	(105−108)
sucrose, glycerol, maltose, maltodextrin, sorbitol, 1,6-anhydroglucose, and trehalose	freeze-dried glass	igG1, monoclonal antibody, cytokines, pepsin, plasma components, trypsin lysozyme and catalase	(109−114)
trehalose	convective air drying	whey protein	(115)
micelles/reverse micelles	zwitterionic surfactants, block copolymers	cytochrome *c*, laccase spore coat protein A	(116,117)

Beyond vitrification in a glassy matrix of an additive such as sugar, methods that entrap or encapsulate proteins in polymer matrices are also used to stabilize proteins. During entrapment, the protein is fixed inside as the matrix that forms around it via polymerization. This process has been used to immobilize proteins at surfaces using sol–gels.^86−95^ Encapsulation by an environment with cavities, such as reverse micelles, often similarly increase protein stability,^96^ although there are exceptions.^97,98^

From sugars in jam to salt in beef jerky and fish, food can be preserved by increasing the environment’s osmotic pressure, thereby dehydrating the food, impeding bacterial growth, and regulating volume, viscosity, and quinary interactions within cells.^99^ Osmotic pressure, Π, is a colligative property that describes the change in water chemical potential due to the presence of solutes, and is reported in units of the equivalent hydrostatic pressure required to exactly negate the change in water activity due to solute addition. This pressure is embodied in the van ’t Hoff equation (earning van ’t Hoff the first Nobel prize in chemistry), often generalized as Π = *iϕCRT*, where *C* is solute concentration, *R* is the gas constant, *T* is temperature, *i* is an index representing the extent of dissociation in solution (e.g., *i* = 2 for NaCl), and ϕ is the osmotic coefficient that describes deviation from ideal behavior, where *ϕ*_*ideal*_ = 1.^100^ Molecular glasses that are almost devoid of water generate exceptionally high osmotic pressures that increase food and drugs shelf life due to their antibacterial effects and their potential to increase protein stability.

In summary, protein stabilization in glassy environments holds significant technological advantages and remains a central strategy. To design more efficient environments tailored for protein stabilization, it will be important to resolve the molecular mechanisms of stabilization. Specifically, it is key to link the properties of the glass-former and glass properties to effects of the glass on protein stability.

### 1.4 Outlook

We focus on recent efforts to resolve the mechanisms by which the glassy state protects dry proteins. We start by describing the properties of molecular glasses most relevant to protein protection. Next, we describe the main methods and proposed mechanisms for stabilizing proteins in glass. We end with open questions and a prospective. Glassy matrices also protect DNA and lipids, and similar strategies are employed in protection from other types of stress, including freezing. These aspects are not covered here, but excellent reviews are available.^10,36,70,118−120^

## 2 NATURE OF MOLECULAR GLASSES

### 2.1 Defining a Glass

**Figure 2:**
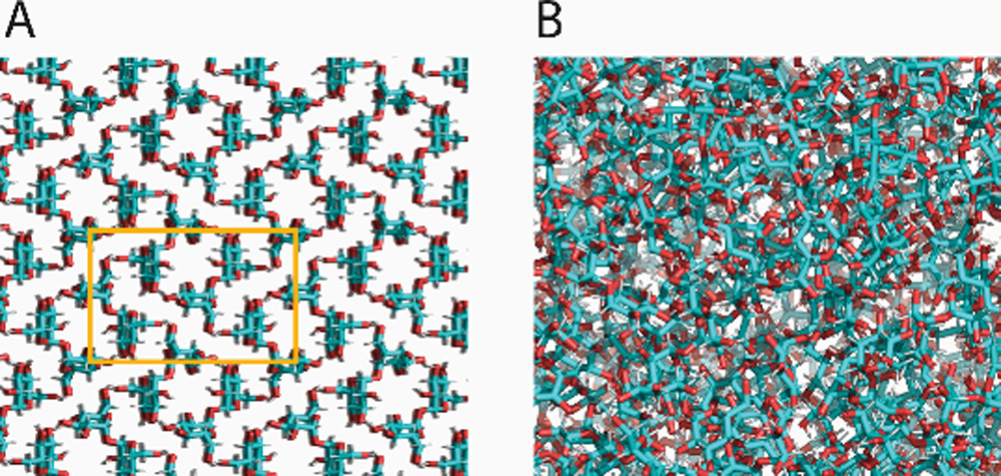
Schematic of the disaccharide trehalose crystal structure (A) and in the glassy state (B). Trehalose’s crystal unit cell is shown as an orange rectangle in panel A, as resolved by Nagase et al.^416^ Trehalose glassy structure is resolved from molecular dynamics simulation.^231^

What distinguishes the glassy state from other states of matter? Unlike a solid crystal, molecular glasses are amorphous. They possess no long-range order, and atomic positions cannot be inferred from a repeating unit cell because no unit cell exists (Figure 2). Even though at the atomic level a glass resembles a supercooled liquid, unlike a simple liquid, glasses exhibit elastic deformation when subject to external forces. However, if an external force is applied slowly, a glass can display inelastic liquid-like deformation over extended periods of time.^121^ Thus, the fundamental nature of a glass depends on the time scale at which it is observed: a glass is essentially a supercooled liquid with such high viscosity that long-range diffusion is unobservable within relevant experimental times.^122,123^ Consequently, a glass behaves like a solid on short time scales and a liquid on (extremely) long time scales.

Many materials act as glass formers, including oxides and sulfides (such as SiO_2_,^124^ BO_3_,^125^ and As_2_S_3_^126^) salts (like ZnCl_2_,^127^ and BeF_2_^128^) alloys (including CuZr^129^) polymers, (including polystyrene,^130^ polyvinyl chloride,^131^ and polycarbonate^132^) and even pure water.^133−135^ Notably, water can be more readily vitrified by adding large amounts of solutes, not only because solutes hinder crystallization of water into ice but also because many solutes are themselves glass formers.^136^ Solutes that facilitate glass formation include salts like LiCl^137,138^ and Ca(NO_3_)_2_;^139,140^ sugars like glucose,^141^ sucrose,^142^ and trehalose;^143^ and polyols such as glycerol,^144,145^ ethylene- and propylene glycols.^146,147^

**Figure 3:**
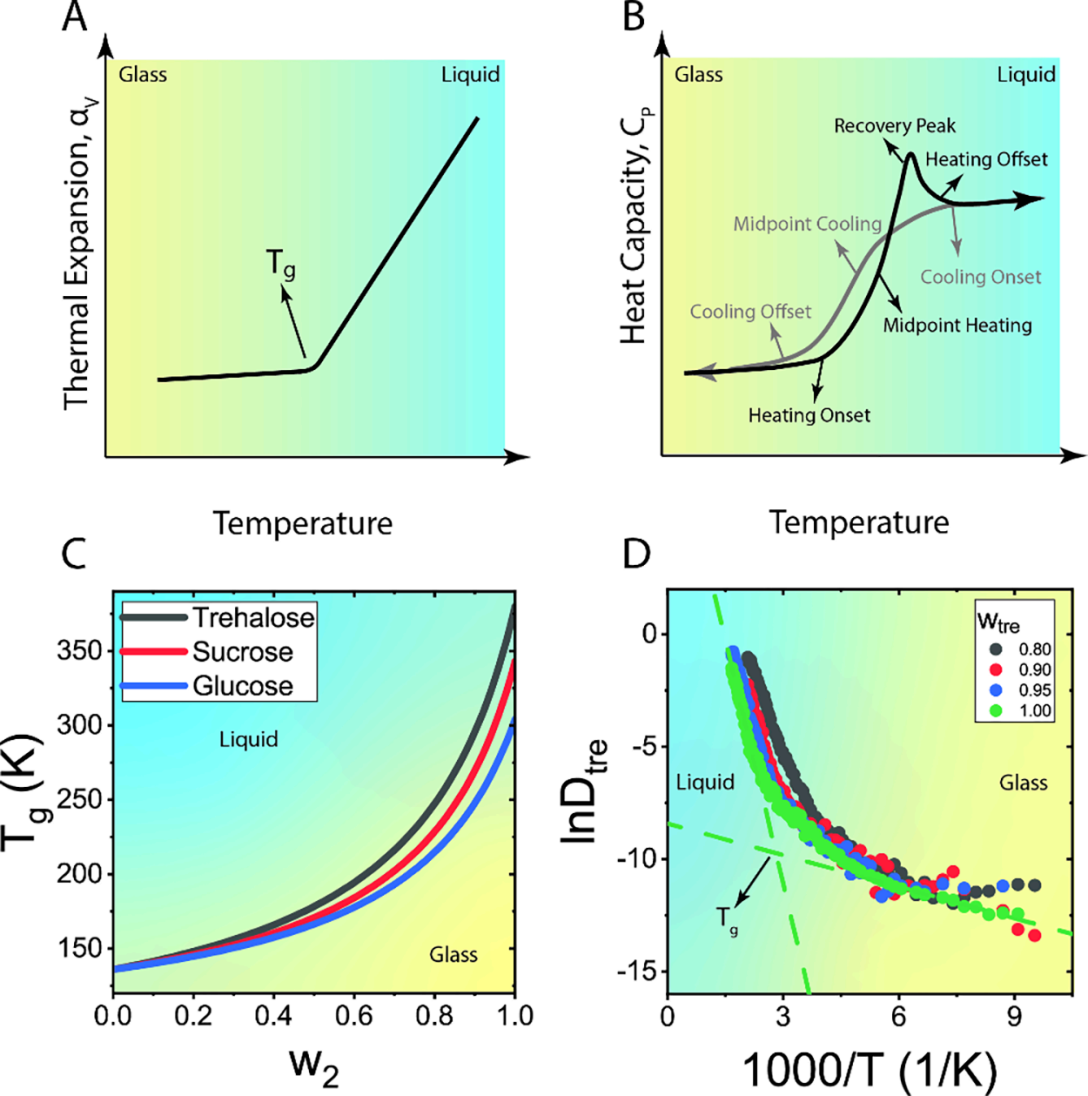
Methods for determining *T*_*g*_. (A) Thermal expansion coefficient, *α*_*V*_, versus temperature. (B) Heat capacity, *C*_*P*_, versus temperature. Heating cycle is in black and cooling cycle in gray. (C) Gordon–Taylor curves for binary aqueous sugar mixtures showing *T*_*g*_ versus sugar weight fraction. For trehalose, sucrose, and glucose, the values of *k* are 4.76, 4.94, and, 4.52, respectively.^417^ (D) Natural logarithm of trehalose diffusion coefficient versus inverse temperature for different trehalose weight fractions as seen in molecular dynamics simulations.^222^ Blue background depicts the liquid regime and yellow depicts the glass.

The glass transition temperature, *T*_*g*_, is an important property of a glass former. *T*_*g*_ characterizes the temperature at which the glass former transitions between supercooled liquid and amorphous glass and reflects a material’s intermolecular interactions. This temperature is readily understood by following a cooling process. In the absence of crystallization, as a glass former cools below its melting temperature, *T*_*m*_, the volume, *V̅*, and entropy, *S̅* (where the macron stands for a molar quantity) of the supercooled liquid gradually decrease. However, once a certain temperature *T*_*g*_ is reached (*T*_*g*_ < *T*_*m*_) the viscosity sharply increases (typically 1000-fold within a 10 K range), while diffusion sharply decreases (Figure 3D).^136^ This high viscosity corresponds to long molecular relaxation processes, making the equilibrium state virtually unattainable. This characteristic implies that the glass transition is a kinetic event, in contrast to crystallization and other first-order phase transitions that are equilibrium minima in free energies, making the glass state metastable. Moreover, the properties of glass, such as *V̅* and *S̅*, exhibit hysteresis,^122^ so that they depend on the method of preparation. For example, more rapid cooling leads to larger values of *V̅* and *S̅* compared to slow cooling because there is less time for relaxation. At the glass transition, the molar volume and entropy begin to deviate from their behavior in the liquid state, as seen in their weaker temperature dependencies in glass. This results in *V̅* and *S̅* values that are larger in the metastable glassy state compared to the thermodynamically stable crystal. This change in temperature dependence is also observed in the heat released or absorbed, *H̅*, during cooling or heating.

Below *T*_*g*_, the energy content is higher in a glass than it would be under equilibrium conditions because the amorphous structure is far from equilibrium. Values of *V̅*, *S̅*, and *H̅* will decrease as the glass “ages” toward its equilibrium state through slow relaxation. Above *T*_*g*_, the material has high molecular mobility and can reach thermal equilibrium. Enthalpic relaxation involves the reduction of the glass enthalpy, *H̅*_*glass*_, toward its value in the crystal, *H̅*_*crystal*_, below *T*_*g*_. In contrast, enthalpic recovery is the enthalpy that the sample releases or dissipates as it relaxes toward equilibrium at the conclusion of a heating cycle, above *T*_*g*_. Enthalpic recovery manifests as a peak in heat capacity, *C_P_* = (∂*H*/∂*T*)_P_ (Figure 3B). This peak originates from surplus heat required to raise the temperature above *T*_*g*_ for a sample that aged (relaxed below *T*_*g*_) toward its equilibrium state.^148−152^ The extra heat can be attributed to the glass transitioning toward a more stable state, somewhat closer to the crystal. A proper dissection of the heat flow due to the heat capacity and enthalpy of relaxation as it passes through *T*_*g*_ requires careful analysis of calorimetric data.^153,154^

Quantifying *T*_*g*_ is challenging. First, the glass transition is a gradual process that can span a wide range of temperatures. Moreover, the range over which the transition occurs varies, depending on experimental methodology or experimental protocol. Finally, as described above, *T*_*g*_ depends on the rate of cooling or heating and the history of the sample, including its preparation and manipulation. Plus, there are important differences between cooling and heating cycles. For example, while cooling cycles exhibit hysteresis only in relation to cooling rates, heating cycles are also influenced by the properties of the preheated glass.

The value of *T*_*g*_ is typically determined by dilatometry^155−158^ or calorimetry.^136^ In dilatometric measurements, *T*_*g*_ is identified by a distinct kink in plots of thermal expansion coefficient, *α*_*V*_ = (∂ ln *V̅*/*∂T*)_*P*_, versus temperature (Figure 3A). In calorimetric measurements, e.g., differential scanning calorimetry (DSC), *T*_*g*_ is determined from the change in the slope of *C*_*P*_ with temperature (Figure 3B). Multiple distinct values of *T*_*g*_ can be gleaned from features in the *C*_*P*_ versus *T* curves of the cooling or heating cycles, specifically the cooling or heating onset, offset, and midpoint, as denoted in Figure 3B.^136,152,159,160^ The midpoint *T*_*g*_ is the most frequently employed value due to the inherent difficulty in precisely pinpointing the onset and offset during the gradual process of the glass transition. Furthermore, two distinct methods, namely the inflection point and the half-step point, can be used to determine the *C*_*P*_ midpoint. Notably, *T*_*g*_ values derived from the midpoints and the temperature of maximal enthalpic recovery are particularly valuable, as they facilitate the calculation of the activation energy associated with relaxation processes in the glassy state.^148^

For mixtures, such as those involving carbohydrate protectants and water, *T*_*g*_ varies considerably with composition. Typically, *T*_*g*_ increases with protectant content because of its higher viscosity, which arrests diffusion and reduces the likelihood of crystallization.^161^ In practical terms, the dependence of *T*_*g*_ on protectant concentration in a mixture is often described by the Fox,^162^eq 1, or Gordon–Taylor, eq 2, equations:^163^
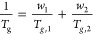
1
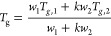
2where *T*_*g*,1_ and *T*_*g*,2_ are the glass transition temperatures of the pure components and *w*_1_, *w*_2_ are the components weight fractions, with *w*_1_ + *w*_2_ = 1. Figure 3C shows examples of Gordon–Taylor curves for sugar–water mixtures that use the additional empirical parameter *k*.

At elevated levels of hydration, *T*_*g*_ of an aqueous mixture can deviate significantly from the predictions made by the Fox and Gordon–Taylor equations due to the formation of crystalline ice. The formation of these ice particles is influenced by the rate of cooling.^164,165^ Slower cooling rates result in the development of larger ice crystals, effectively dehydrating the amorphous mixture as glass-forming components are typically expelled from the crystalline phase. Consequently, the mixture exhibits two distinct phases: crystalline ice and a concentrated amorphous glass. During a heating cycle, this mixture that contains ice and glass, will produce two calorimetric signals, one for the glass to liquid transition and second for the melting of crystalline ice.^164^ The glass transition temperature of the concentrated glass, *T_g_*′, is larger than *T*_*g*_ of a pure glass mixture because the higher glass former concentration also leads to higher *T*_*g*_. The melting temperature of ice crystals that are embedded in the glassy environment, *T_m_*′, is lower than that of ice in pure water, *T*_*m*_, because of the increased entropy associated with water–protectant mixing in the glass. Increasing the cooling rate reduces the size of the ice crystals so that when cooling is fast enough, the purely glassy behavior (in absence of any crystalline ice) described by eqs 1 and 2 is reestablished.

**Figure 4:**
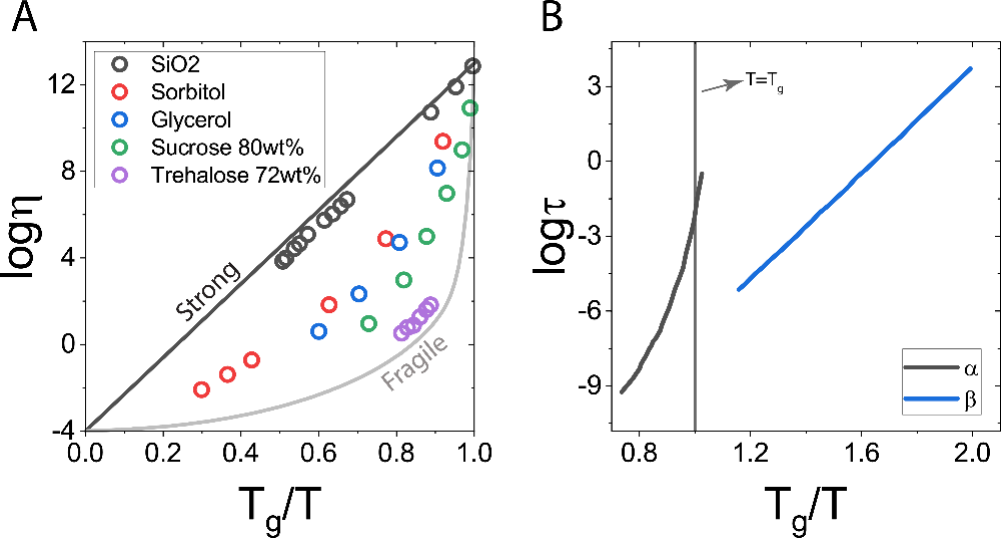
Strong and fragile glasses. (A) Angell plots of SiO_2_, sorbitol, glycerol, sucrose, and trehalose aqueous mixtures from viscosity measurements. Adapted with permission from ref (418), copyright 1997, Springer Nature, and ref (136), copyright 2002, American Chemical Society. Black curve is for strong glass former, gray is for fragile. The curves of strong and fragile glass formers typically intersect at *T* = *T*_*g*_ because *T*_*g*_ is usually taken as the temperature where log η = 13 (in poise) or where *τ*_*α*_ = 100 s, which for many materials is close to *T*_*g*_, as derived from dilatometry or calorimetry measurements.^205^ By contrast, β-relaxation is considerably faster, often on the order of 1 *μs* at *T*_*g*_. (B) Angell plot of α- and β-relaxation for polyethylene terephthalate. Adapted with permission from ref (419), copyright 2006, John Wiley & Sons. *T* = *T*_*g*_ is marked by a vertical line.

Glass formers are also categorized by their fragility, defined as the extent of deviation of viscosity from Arrhenius behavior.^123,136^ This fragility has little to do with the brittleness of the glass. Instead, fragility characterizes the viscosity of the liquid as it approaches its glass transition. The glass former fragility can be readily inferred from the Angell plots^123,166^ showing the logarithm of the viscosity, logη, versus *T*_*g*_/*T* (Figure 4A). Scaling *T* by *T*_*g*_ in the Angell plot allows the comparison of glass formers that undergo the glass transformation at different temperatures. Below *T*_*g*_, a “strong” liquid adheres to an Arrhenius relationship, while a “fragile” liquid exhibits super-Arrhenius behavior, resulting in a sharp upswing in viscosity as *T*_*g*_ is approached. The super-Arrhenius behavior of fragile glass formers implies that the energy barrier for molecular relaxation increases as the temperature is lowered toward *T*_*g*_, and subsequently that the molecular motion becomes progressively more cooperative as temperature decreases.^167^

Figure 4A shows examples of strong and fragile glass formers. For example, SiO_2_ is a strong glass former, whereas molecular mixtures and melts, such as those composed of polyols, sugars, and other organic compounds with or without water, tend to be fragile. It is useful to rank fragility by the steepness or fragility index, *m*, describing the change in viscosity as *T*_*g*_ is approached:^168,169^
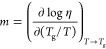
3A larger value of *m* corresponds to a more fragile glass former. For example, the fragile anhydrous trehalose has *m* = 107, while for the less fragile (stronger) glycerol *m* = 47, and for the strong SiO_2_*m* = 20.^170−172^

The fragility of a glass former is also related to its α-relaxation time, *τ*_*α*_, which characterizes the continuous evolution of the structure of the liquid or metastable glass. There are different types of α-relaxation times that depend on the method of measurement, including shear (*τ*_*S*_),^173−177^ dielectric (*τ*_*D*_),^178,179^ spin (*τ*_*C*_),^179−181^ and enthalpy (*τ*_*H*_)^182,183^ relaxation times. These relaxation times are related to each other through the complex motions of the glassy matrix.

Like viscosity, each relaxation time serves as a means to assess liquid fragility based on its departure from Arrhenius behavior. These relaxation times are indicative of macroscopic motions that are progressively more constricted as the temperature drops and the supercooled liquid vitrifies. However, distinct relaxation times may manifest slightly different temperature-dependecies.^184^ Note that using *τ*_*S*_ in Angell plots is equivalent to using η since according to Maxwell’s relation the two are linearly dependent.^185^

For many glass formers, a second relaxation process, called the Johari–Goldstein or β-relaxation, emerges below *T*_*g*_.^186−189^ The corresponding β-relaxation time, *τ*_*β*_, in a fragile glass exhibits Arrhenius dependence below *T*_*g*_ (Figure 4B). α-relaxation is several orders of magnitude slower (Figure 4B). The difference in the two time scales increases with decreasing temperature. However, the extrapolation of α- and β-relaxation suggests they converge at high temperatures.^190^ Moreover, the activation energy for β-relaxation is smaller than that of α-relaxation. For example, for a 43.8 mol % toluene-pyridine glass at −140 °C, the activation energies are 50 and 5.4 kcal/mol for α- and β- relaxation, respectively.^189^

**Figure 5:**
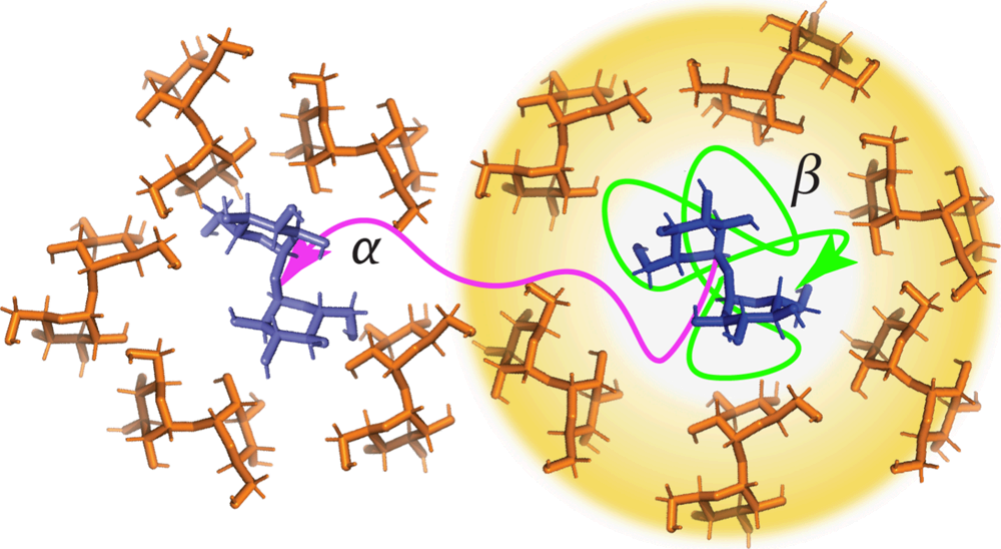
Schematic of motions associated with α- and β-relaxations. β-relaxation involves limited “rattle” movements of molecules confined by neighboring molecules in the glass, while α-relaxation is related to larger motions that contribute to the ongoing changes in the glass structure. See text for details.

Although the precise origin of β-relaxation is unknown, it is generally considered to encompass the motion of an entire molecule or some larger repeat unit (e.g., for a polymer glass former).^191^ This process is typically associated with the reconfiguration of molecules “rattling” within the cages formed by neighboring molecules in the vitrified state, as well as shorter events involving the breaking of these cages.^192^ Because β-relaxation involves rapid and rigid movement of molecules (or repeat units), some argue that this shorter relaxation is a precursor to the α-relaxation and that these faster motions are essential for the viscous flow and diffusion responsible for the longer α-relaxation (Figure 5).^193^ Regardless of the argument’s validity, *τ*_*α*_ and *τ*_*β*_ are correlated in terms of their pressure and aging dependencies, and tend to merge at elevated temperatures above *T*_*g*_, suggesting that the processes are coupled.^190,191,194^

### 2.2 Theory of Glasses

So far, we have described the properties of a glass and the phenomenological parameters to characterize them. We now summarize some theoretical efforts to model the molecular origins of these properties. Initially, most of these efforts were directed to modeling the dependence of the viscosity, and consequently τ, on temperature in supercooled states. The simplest model, suggested by Andrade, describes an Arrhenius-like dependence of η on temperature,^195^
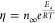
4where *E*_*a*_ is the activation energy and η_∞_ the limiting viscosity value as *T* approaches infinity. Developments by Volgel,^196^ Flucher,^197^ and Tammann and Hesse^198^ led to the Volge–Flucher–Tammann (VFT) equation,^199^
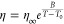
5with *T*_0_ the temperature where the viscosity diverges, and *B* is an empirical parameter. *B* was later related to the fragility through *B* = *DT*_0_, with the empirical parameter *D* < 20 corresponding to a fragile glass former and *D* > 100 corresponding to strong glass former.^136,200^ Further improvement to the temperature dependence of the viscosity is given by the Williams–Landel–Ferry (WLF) equation.^201^ The dependence of viscosity on external pressure can be modeled by an analog of the VFT equation,
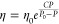
6where *P*_0_ is the idealized glass transition pressure, η_0_ is the extrapolated zero pressure viscosity and *C* is the empirical pressure fragility parameter. More complex models for the pressure dependence have also been proposed.^200,202^

Two important theoretical descriptors of mobility in glass and glass viscosity are the free volume and the excess configurational entropy. Under the free volume framework suggested by Doolittle,^203,204^ the viscosity increases upon cooling because of the reduced space afforded to each particle. The viscosity then depends on the free volume, *V*_*f*_, defined as the volume that arises due to thermal expansion of the glass, so that *V*_*f*_ = *V* – *V_oc_* with *V* the total volume and *V_oc_* the occupied volume. The dependence of viscosity on volume is then:^175,176^
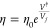
7where *V*^†^ is a fitting parameter.

Excess configurational entropy, *S*_*c*_, is associated with the diversity of configurations of the supercooled liquid particles with respect to the crystal. When extrapolated to below *T*_*g*_, *S*_*c*_ approaches zero at the so-called Kauzmann temperature, *T*_*K*_, suggesting that below *T*_*K*_ the entropy of the metastable state is less than the entropy of the crystal.^205^ In practice, this inversion of entropy is avoided by the purely kinetic glass transition, implying, paradoxically, that at some (long) time such an inversion could be attained.^206^ For many glass formers the value of the thermodynamic *T*_*K*_ and kinetic *T*_0_ coincide, which might explain the apparent viscosity divergence of the VFT model (eq 5).^207^

Kauzmann attempted to resolve the paradox by suggesting the crystallization of the metastable state between *T*_*g*_ and *T*_*K*_.^205^ Despite its plausibility, Kauzmann’s resolution has not been corroborated experimentally, and subsequently, the “Kauzmann paradox” drove the development of new theories. In their work, Gibbs and DiMarzio (GD), developed a lattice theory suggesting the onset of a second-order phase transition upon cooling above or at *T*_*K*_.^208^ Building on GD theory and by using detailed balance arguments, DiMarzio and Yang developed a kinetic theory relating particle trapping and escaping from deep wells to glass properties. These properties include the viscosity, which unlike the VFT theory does not diverge at any temperature, circumventing the Kauzmann paradox.^209^ The dynamic nature of supercooled liquid and glass, including caging and transitions between metastable states, is further emphasized in the fluidized domain model,^210,211^ mode coupling theory,^212−215^ random first order transition theory,^207,216^ and the self-consistent generalized Langevin equation.^217^

The influence of thermal history and aging on glass energetics can be analyzed using the Tool–Narayanaswamy–Moynihan (TNM) model. TNM is particularly valuable because it can be applied to fit differential scanning calorimetry (DSC) experiments, accommodating various scanning protocols, rates, aging times, and temperatures. Through TNM fits, insights are gained into the activation energy governing relaxation processes and the correlation between enthalpic recovery and sample history.^148^ To address the nonexponential nature of relaxation processes in glass, TNM introduces an empirical parameter 0 < β̃ ≤ 1, denoting the deviation from exponential behavior (the tilde distinguishes it from β-relaxation). The relaxation decay function is then expressed as^218^
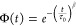
8Here, τ_0_ represents the characteristic relaxation time that is given by
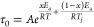
9where *E*_*a*_ is the activation energy, *x* is the nonlinearity parameter, and *T*_*f*_ is the fictive temperature signifying the temperature at which the measured property (e.g., volume or enthalpy) would reach equilibrium. To streamline the extraction of activation energies and pre-exponential factors for relaxation processes from constant rate DSC experiments, a minimal model that encompasses enthalpic relaxation and recovery has been proposed.^219^

**Figure 6:**
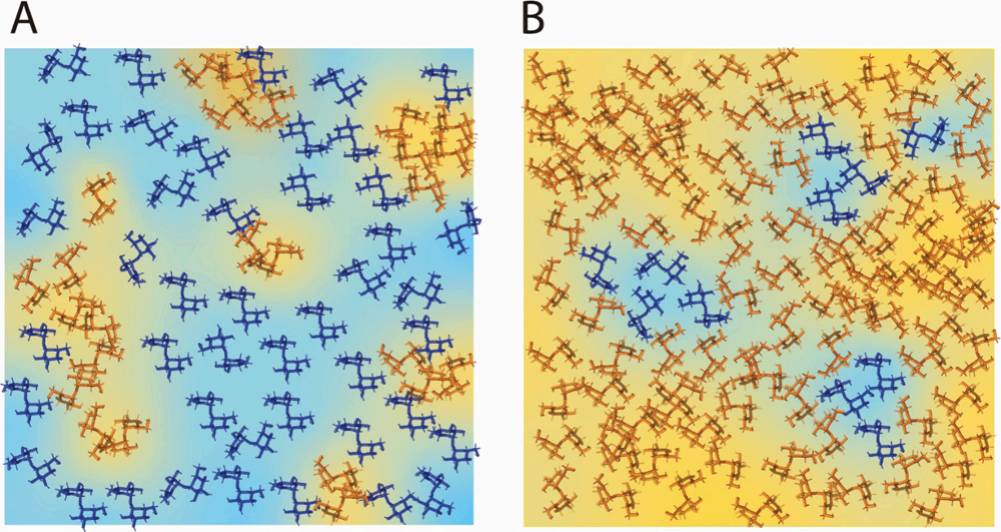
Schematic of cooperatively rearranging regions (CRRs) above *T*_*g*_ (A) and in a molecular glass (B). The glassy CRRs are shown in orange, liquid regions in blue.

An alternative molecular mechanism was suggested by Adam and Gibbs (AG).^220^ In their model, the properties of glass are determined by the equilibrium size distribution of cooperatively rearranging regions (CRRs), defined as the smallest region that can change configuration independent of its environment (Figure 6). CRRs and cooperative motion in glass are observed in simulations.^221−225^ The idea is that the size of the CRRs increases upon cooling to comprise the entire glass at *T*_*K*_. By relating the size of the CRRs to *S*_*c*_, the AG model relates the values of *T*_*g*_ and *T*_*K*_, and resolves the temperature dependence of viscosity through
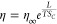
10where *L* is a fit parameter.

CRRs have been further developed in Ginzburg’s “two-state, two (time) scale” model,^190^ which employs a Flory–Huggins type lattice model, where the sites are occupied by two types of elements, a liquid- and solid-like CRRs. Unlike the AG model, the size of these CRRs does not vary with temperature. Instead, the fraction of solid CRRs, ψ, increases upon cooling, with ψ = 1 corresponding to a lattice of only solid elements. The particle motions in the liquid and solid CRRs follow an Arrhenius behavior with a liquid activation energy, *E*_*l*_, and solid activation energy, *E*_*s*_. By associating the fast β-relaxation with local motion in the liquid CRRs and the slow α-relaxation with the effective motion in both liquid and solid CRRs, the relaxation times are
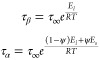
11Here, ψ is determined for a given pressure and temperature by minimizing the lattice model free energy. This simple framework describes the pressure and temperature dependence of both relaxation time scales, including the super-Arrhenius behavior of *τ*_*α*_, and the variation of *T*_*g*_ with degree of polymerization in polymeric glass formers. Further modification allows the model to capture the effect of cooling rate and thickness of a thin glass film on *τ*_*α*_ and *T*_*g*_.^226,227^

To date, the predominant focus in theoretical research has been on simple glass formers, characterized as spherical particles that interact primarily through steric interactions,^190,202,212,213,215,217^ with occasional consideration of close-range interactions.^214,226−228^ These models thus overlook the intricate structural characteristics of most real glass formers. While this simplification facilitates the generalization of properties concerning the α- and β-relaxation processes, including their temperature and pressure dependencies, it can hardly resolve the implications of more complex structure and intermolecular interactions (such as hydrogen bonding and van der Waals forces) to both relaxation processes.

## 3 PROTECTING PROTEINS UNDER CONDITIONS OF LIMITED HYDRATION

Anyone who has prepared glass candy knows molecular glasses are easy to make: sucrose and water are simply combined at high temperature and then cooled.^229^ Glucose is added to prevent sugar recrystallization, probably by enhancing the impact of mixing entropy or increasing packing frustration of the nonhomogeneous mixture. The result is a transparent and brittle glass. If exposed for too long, sorption of ambient water to this hygroscopic medium damages the glass. In the following sections we discuss the generalizability of the glass formation process. Are details of the glass forming process important in determining the properties of the glass? What are the key considerations when embedding proteins in glasses or aerogels?

### 3.1 Molecular Glasses

The glassy state is reached for molecular glass formers when a liquid is supercooled fast enough below *T*_*m*_ to prevent crystallization (Section 2.1). This quenching process typically involves simultaneous changes in temperature and hydration. Desiccation and temperature quenching are thus coupled in glass formation because the melting point of the mixture depends on hydration.

**Figure 7:**
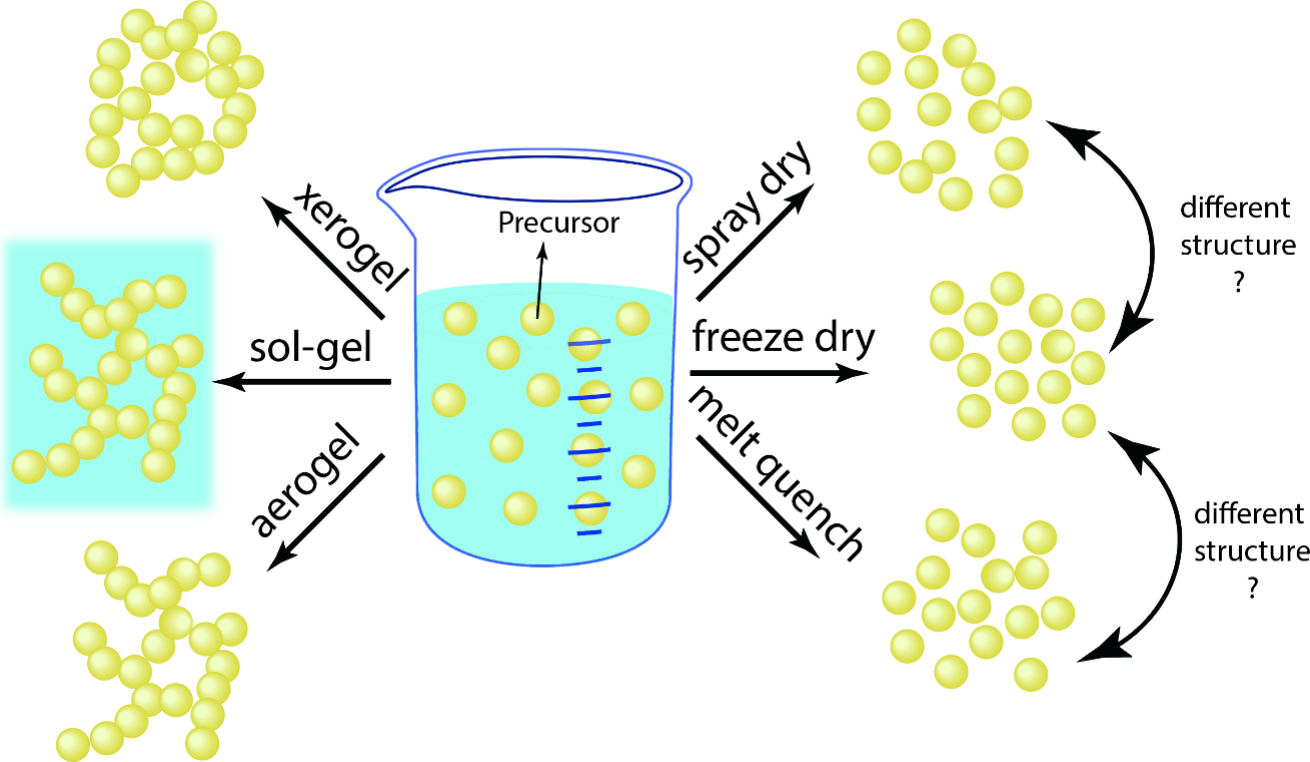
Schematic of methods for forming molecular glasses and gels. Sol–gels, aerogels, and xerogels have controllable distinct morphologies, while the differences in the structure of molecular glasses formed using different methods are not as well resolved (Section 3.1).

Glass is metastable. Therefore, its structure and properties depend on the method and conditions of preparation as well as the history of the sample once it is formed.^123^ Specifically, glasses may be quenched to states with different local structures and order, which may modify processes within the glass, including water sorption and crystallization, that directly impact properties including viscosity, glass transition temperature, and relaxation. This diversity complicates the technological application of preservation by glass in the pharma and food industries because glass can be unpredictable. Lack of predictability has led to a search for robust preparation methods. Four distinct methods have been reported for creating amorphous sugar glass with little or no water. These methods are freeze-drying, spray-drying, vacuum drying, and melt quenching (Figure 7),^230^ although many variations have been reported.^231,232^

Freeze-drying, or lyophilization, involves freezing of an aqueous solution of sugar or other glass former (typically at concentrations of 0.25–1 M) well below the freezing point (typically −45 to −200°*C*), with subsequent drying at low pressure (less than ∼100 Torr). The initial solution may also include proteins or other molecules of interest for embedding in the glass. The fundamental idea is to exploit the slow protein dynamics in the quenched cold state and the low vapor pressure of water under vacuum, so that by reducing the pressure, water is removed as vapor by sublimation. This process unfolds in three steps.^233,234^ First, during the initial freezing, the degree of water crystallization and ice morphology is determined based on factors like cooling rate and glass-former concentration (Section 2.1). In the second step, referred to as primary drying, ice is removed through sublimation. The final step involves secondary drying, during which water trapped in the amorphous phase is evaporated, typically at higher temperatures (e.g., 40–60°*C*) to accelerate water evaporation.^233^ Evaporation rates in both drying stages are controlled by ice formation that occurs during the initial freezing.^165^ Since crystalline ice is a “lousy solvent” for many solutes, including proteins, freezing of pure water may involve these solutes’ exclusion resulting in effective dehydration,^164^ which may lead to protein aggregation. However, the concurrent increase in the concentration of the glass-forming agent, which in turn promotes protein stability in solution and within the glass, protects proteins from misfolding and aggregation.^234^ Importantly, a rapid freezing process impedes harmful water crystallization, affording the entrapment of proteins and other macromolecules in an innocuous matrix of supercooled water. The restricted motions at low temperatures protect the embedded protein (or other macromolecule) during vitrification.

Spray-drying involves spraying an aqueous solution of the glass former into air or nitrogen gas at elevated temperature, typically close to 100°*C*, leading to water evaporation. Spraying through a narrow nozzle produces a powder of finely tuned particle size distribution and morphology. However, the elevated temperature can damage many biomacromolecules.^235^ In the vacuum drying method, the solid glass former is dehydrated by heating under vacuum for several hours, at temperatures less than 100°*C* and pressures under 100 Torr. Nitrogen flow may be used to exclude oxygen and water vapor. Melt-quenching typically involves briefly heating the solid glass former well above the glass forming temperature under nitrogen gas flow, followed by rapidly cooling, e.g. by plunging into liquid nitrogen. The resulting glass in all these methodologies may be further milled to obtain flowing powders for technical applications.

Heating must be considered carefully because it promotes unwanted chemical reactions of both glass former and protein. In particular, sugars can undergo Maillard and caramelization reactions causing nonenzymatic browning.^236^ In the Maillard reaction, reducing sugars and amino acids react at moderate temperatures to produce melanoidins. Nonreducing sugars such as trehalose do not undergo this reaction. Caramelization typically proceeds at higher temperatures, and only sugar, reducing or nonreducing, reacts. The products are polymers, including caramelans, caramelens and caramelins.

Maillard and caramelization reactions are detrimental to both glass formation and embedded proteins. Thus, nonreducing sugars and ones that resist caramelizing will be easier to manipulate in technological applications, making trehalose an attractive choice.^237^ Its inertness may in part explain why trehalose is selected by so many organisms as the glass-former of choice in combating desiccation.^238^ Beyond chemical reactivity, heating can also induce protein unfolding and denaturation. Heating proteins close to and above their melting temperature should thus be avoided. However, the stabilization that sugars impart on proteins, even in the solution before the formation of glass, may circumvent the deleterious effect of solution heating en route to glass formation.^5,239−242^

Sample preparation history can impact glass properties. In a study of trehalose glass formation, several properties were compared using different preparation methods.^230^ Interestingly, fragility, transition temperature, and the heat capacity change at the transition temperature varied only slightly between methods, but the onset temperature of crystallization and enthalpic relaxation were notably different. In addition, the method of preparation affected the rate and extent of water sorption, and sorption removed the effects of structural history in the amorphous dehydrated phase.

In another study, the preparation method influenced the amount of water in trehalose glass.^232^ Water content is important because it can affect the mode of protein protection, as well as many glass properties, including *T*_*g*_ (Figure 3C). In addition, the concentration of sugar in the initial solution from which the glass is prepared influenced the outcome of the preparation: high concentrations of sugar or large volumes led preferentially to formation of the trehalose dihydrate crystal, while lower concentration or volumes more often resulted in a glass.^232^ Trehalose glass may tolerate significant hydration while maintaining a high *T*_*g*_ (close to 100°*C*), perhaps because excess water tends to form the dihydrate crystal, which separates from the glass.^243−245^ The removal of water from the glassy state by the dihydrate crystal may be another reason trehalose was selected by evolution for desiccation protection.

Adding components to the glassy matrix can improve the stability and function of embedded proteins. Mixing polyethylene glycol (PEG) with sugar alcohols or sugars (trehalose, lactose, or mannitol) in a freeze-dried matrix preserves lactate dehydrogenase or phosphofructokinase better than the respective single-component matrices.^246^ In other studies, adding glycerol to a sugar glass matrix improved its ability to protect proteins.^247,248^

### 3.2 Solgels, Aerogels, and Reverse Micelles

A sol–gel process involves an inorganic collodial suspension that forms a three-dimensional gel (Figure 7).^249^ In contrast to molecular glasses that form a matrix through noncovalent bonds, sol–gels processes entrap proteins by creating a chemically bonded network. Sol–gel syntheses immobilize proteins by encapsulating and adsorbing them into the gel’s porous structure.^103^ This strategy is considered “hard entrapment” in contrast to the “soft entrapment” by molecular glasses.^250^ For several decades, immobilization and entrapment of proteins and other biologically relevant molecules through the sol–gel processes has presented itself as an attractive methodology for enzyme applications and biosensor preparations.^101,250−252^ Entrapping enzymes in these gels has evolved into one of the most popular ways to immobilize enzymes, with wide ranging applications in biotechnology and biomedicine, and for environmental, synthetic, and sensing uses.^253−259^

The chemical process involved in forming the oxide matrix is simple, and is achieved under mild conditions, including low temperature and moderate pH. In addition, the porosity and chemical nature (such as hydrophobicity vs hydrophilicity) of the matrix can be tuned. Another advantage is that a significant amount of water can be maintained in the matrix, which facilitates protein activity.^260,261^

The sol–gel process is based on the ability to form silica, metaloxide, and organosiloxane matrices of defined porosity by the reaction of organic precursors at room temperature.^228,262^ Enzyme immobilization is typically achieved through a polymeric (or alkoxide) route. First, oxide precursors are suspended or dissolved, usually at acidic pH in the presence of water. For silica-based sol–gel syntheses, the matrix precursors are tetraalkoxysilanes or mono-, di-, or trialkyl alkoxysilanes, most commonly tetramethoxysilane (TMOS) and tetraethoxysilane (TEOS).^250^ In the next step, condensation reactions between silanol moieties are promoted by activating the hydrolyzed precursor with a base (e.g., KOH). Condensation results in a siloxane polymer matrix that grows until the onset of gelation. Proteins can be trapped in the resulting matrix as it forms around them.^101,263−265^ Moreover, enzyme activity is often preserved in the matrix.

Sol–gel synthesis can be detrimental to protein stability. Alcohols (ethanol and methanol) released from TMOS and TEOS as byproducts of hydrolysis and condensation reactions can harm protein activity, although they are less damaging than long-chain alcohols.^101,250,266,267^ Moreover, sol–gel synthesis requires harsh conditions, such as hot concentrated KOH or HF, to release the proteins. Alternative sol–gel precursors such as poly(glyceryl silicate) help alleviate this problem.^268^ Recently, Potnis et al. reported a gentle release method that avoids strong acids and bases via their “Capture and Release Gels for Optimized Storage of Biospecimens System”,^103^ which avoids the damaging effects of acids, bases, and alcohols on proteins by using a short microwave treatment for hydrolysis of TMOS and then rotary evaporation of methanol from the solution of orthosilicic acid.^103^

Parameters for gel formation, including pore size and entrapment tightness, as well as pore-wall properties, can be controlled by adjusting the ratio of H_2_O to Si, pH, and solvent.^269^ A low pH and low H_2_O:Si ratio favor gel tightness, with smaller pores generated by a cluster–cluster polymerization, within a highly branched matrix. Under alkaline conditions, condensation is limited by the availability of hydrolyzed precursors, and polymer growth is driven by a monomer-cluster process, resulting in a looser silica matrix.^262,269,270^ Increasing gel tightness can block or slow conformational transitions necessary to achieve enzyme function, resulting in protein conformations with higher or lower activity. As for pore surface properties, NMR data show that even though the gel pores are hydrophobic, the presence of some free hydrophilic groups inside the pore, like hydroxyl groups from hybrid gel formed by TMOS/*n*-butyltrimethoxysilane precursors, has a role in slowing exchange of polar solvents, preserving enzyme activity.^250^

While gels formed by sol–gel synthesis often provide an excellent matrix for proteins, they are considerably hydrated, and developing ways to preserve proteins in the dry state are desirable. Indeed, the hydrated gels can be desiccated to produce solvent-free xerogels or aerogels that can hold proteins or other guest molecules. While both types of gels are formed by drying the hydrated gel, xerogels are realized by dehydrating liquid from the sol–gel, typically at, or close to, ambient conditions, while aerogels are achieved either by freezing with subsequent solvent extraction at low pressures through sublimation, or by removing the solvent at supercritical temperatures and pressures. The aerogel process is gentler, preventing capillary forces from acting on the sol–gel matrix as solvent is removed, leaving the matrix largely intact and highly voluminous.

Aerogels and xerogels differ in many ways. Perhaps most notably, aerogels shrink less upon drying, which leads to greater porosity, specific surface area, and a lower bulk density.^95,271,272^ In fact, aerogels hold the record for the lowest density solid.^273^ Protein can be trapped in silica aerogels by first forming the hydrated gel around the protein in solution and then replacing water with ethanol with subsequent critical-point drying in an atmosphere of CO_2_. Enzymes can be trapped in aerogels, typically at 35 to 40°*C*, without denaturation, exceeding the activity of proteins embedded in the more traditional xerogels.^95^ Thermal stabilization is also seen, and large substrates inaccessible to the enzymes in xerogels may be accessible in aerogels.

Both hydrogels and aerogels can also be realized using other matrix elements. For instance, proteins can be used as scaffolds for other protein or drug molecules in the aerogel formation process.^274^ Protein aerogels are advantageous due to the ability to control the surface area that can reach up to hundreds of square meters per gram, and to their exceptional porosity, enabling efficient drug intake, making them suitable for drug delivery. Furthermore, their low densities, typically between 0.003 and 0.5 g/cm^3^,^275^ are advantageous for drug delivery systems, particularly for medications administered mucosally, such as through oral or nasal routes.^276^ Milk or egg proteins are popular starting materials, as well as gelatin and elastin. Carbohydrates can also be used as matrix elements, including cellulose, chitosan, and hyaluronic acid.^277^ Proteins designed to combat desiccation can also form aerogels. CAHS D aerogels retain the structural units of their hydrogels, but the details depend on prelyophilization concentrations. Only at higher concentrations do slabs form that then comprise the walls of the aerogel pores. These changes in morphology are associated with a loss in disorder and an increase in large β sheets and a decrease in α helices and random coils.^60^ In summary, methods of gel preparation are important for determining gel properties, complicating the task of unraveling the mechanisms of protein stabilization in these complex matrices.

Nevertheless, encapsulation and protein restriction can even be detrimental to stability. In fact, in technological applications, entrapment in silica glass matrices often reduces the protein catalytic activity and may destabilize them relative to buffer.^101^ Moreover, in two studies, the interactions of proteins with the confining cavity of reverse micelles destabilized the protein.^97,98^ We discuss mechanisms of protein stabilization and destabilization caused by volume restriction in Section 4.4.

## 4 STABILIZATION MECHANISMS IN MOLECULAR GLASSES AND GELS

### 4.1 Crowding

To lay the groundwork, it is valuable to examine the impact of solute additives or cosolutes on protein stability in aqueous solutions in the liquid state. Perhaps, to a first approximation, the glassy state can be thought of as a highly concentrated solution. In aqueous solutions, protein stability is affected by forces facilitated by added crowders and their preferential inclusion or exclusion from the protein surface. Crowders can assume various forms, including macromolecules (such as proteins or DNA), large polymers, or smaller solutes, also referred to as cosolutes. Crowding, therefore, collectively refers here to the impact of a rising concentration of crowders on stability, structure, association, and aggregation of macromolecules, e.g., proteins.^278−282^

Cosolute inclusion or exclusion is quantified using the preferential hydration coefficient, Γ_*S*_, which captures the average excess or deficit of solvent molecules adjacent to the protein compared to bulk solvent.^231,283−286^ If a cosolute is excluded from a protein’s surface, excess water remains in the protein’s vicinity, that is, the protein is preferentially hydrated, and Γ_*S*_ is positive. Conversely, preferentially included cosolutes result in negative Γ_*S*_. Preferential hydration can be determined from the difference between osmotic pressure of mixtures measured in the absence and presence of protein.^283,287−290^

Importantly, the extent to which a cosolute stabilizes a protein is characterized by the change in Γ_*S*_ upon folding, ΔΓ_*S*_ = Γ_*s*_^*native*^ – Γ_*s*_^*denatured*^. This parameter directly correlates with the change in folding free energy as the protein is transferred from water to a cosolute-containing solution. If a cosolute is more excluded from the denatured state than it is from the native state, ΔΓ_*S*_ is negative and the protein is stabilized (lower folding free energy). By contrast, denaturants (e.g., urea) are preferentially included at the denatured protein state more than at the native state. ΔΓ_*S*_ is therefore positive and the protein is destabilized (higher folding free energy).^283,291−293^ For example, for the β-hairpin model miniprotein MET16 ΔΓ_*S*_ is ca. −42 for the strongly stabilizing disaccharide trehalose over a wide range of concentrations, but only −23 for its moderately stabilizing monomer glucose.^239,241^ The same ΔΓ_*S*_ is directly proportional to (minus) the m-value, defined as the change in folding free energy with cosolute concentration.^283,294−296^ For example, for MET16, the m-value is approximately 2.4 kJ mol^–1^ M^–1^ in the presence of trehalose and 1.1 kJ mol^–1^ M^–1^ in the presence of glucose.^239,241^ These differences correspond to a respective increase in the folded-to-unfolded ratio by a factor of approximately 2.6 and 1.6 for 1 M sugar. A similar trend in stabilizing effect has been noted for larger globular proteins, e.g., for equine cytochrome c the m-values are 10 kJ mol^–1^ M^–1^ for trehalose and 6.3 kJ mol^–1^ M^–1^ for glucose.^297^

The value of ΔΓ_*S*_, whether negative (stabilizing) or positive (destabilizing), arises from the interplay of interactions between all solution components: protein, cosolute, and solvent. Protein–cosolute interactions include mutual steric excluded volume or hard-core repulsions, and soft (chemical) interactions. Excluded volume interactions are purely repulsive and therefore restrict the volume available to the protein in solution. As the cosolute volume fraction increases there is less space for the protein, forcing it into its most compact state, which, in the presence of water, is most often the native, biologically active, folded state.^298^ The stabilization exerted by excluded volume interactions is further modulated by solvent–cosolute nonideal interactions that impact solution osmotic pressure, Π. The presence of solvent–cosolute attractions (and correspondingly effective repulsive cosolute–cosolute interactions) increase Π for a given concentration. For cosolutes excluded from protein surfaces, this added osmotic pressure further destabilizes any protein exposed interface, thereby favoring compaction. Conversely, solvent–cosolute repulsions reduce Π and thus reduce the stabilizing effect of the cosolute.^241,299^

Protein–cosolute soft interactions can be stabilizing or destabilizing.^5,282,300−306^ If the soft interactions are net repulsive the “effective” volume excluded by cosolutes increases, reinforcing the stabilizing effect. If they are attractive, the exposure of sites buried in the native state is favored, so that more cosolute–protein contacts can become available, making these attractive protein–cosolute interactions destabilizing. Soft interaction depends not only on the cosolute but also on the chemical character of the exposed protein interface. For instance, trehalose exhibits attractive soft interactions with the α-helical model protein AQ16 but repulsive interactions with the β-hairpin model protein MET16.^241^ These contrasting interactions are manifested in the contributions of soft interactions to the m-values: negative for AQ16 (−0.41 kJ mol^–1^ M^–1^) but positive for MET16 (0.22 kJ mol^–1^ M^–1^).^241^ Interestingly, attractive interactions might explain why dry proteins are often destabilized in the absence of a protectant, because the only chemical interactions available to a dry protein involve the protein itself. That is, proteins contain many attractive interactions, and unfolded proteins provide even more attractive interactions than do native proteins. Thus, adding molecules to block or prevent these interactions should lead to more proteins in the native state.

An often-neglected theme impacting stability is the nonideal cosolute–solvent chemical interaction,^307^ which stems from the difference between solution compositions at the protein interface and in bulk solution. Along with the release of cosolute and solvent on folding, the mixing of these liberated molecules with the differently concentrated bulk phase can impact the protein folding free energy.^241^ These nonideal mixing interactions generate heat that can show up as a stabilizing enthalpic contribution,^299^ and is observed for many proteins, particularly in the presence of low molecular weight cosolutes.^239,240,282,308−312^

Much effort has been invested to model and predict the effect of cosolute-protein–water interactions. Excluded volume interactions were first modeled by Asakura and Oosawa^313,314^ and later by using scaled particle theory.^301,315−320^ Soft interactions are usually addressed using weak protein–cosolute binding terms,^302,306,321^ yet newer models also consider repulsive soft interactions^242,307,322^ and even explicitly incorporate cosolute–solvent intractions.^299,307,323^

How far does the analogy of crowding carry to proteins in a glassy matrix? The first hurdle is that there is little or no water in the glassy state, so a picture of a cosolute solvated within a solvent is blurred or even completely inverted (i.e., the cosolute becomes the solvent). Another difference is that the glassy state is amazingly viscous (Section 2.1), so the system may not sample all states on the time scale of the observation. Can we even speak of a well-defined folding free energy? Nevertheless, the image of a protein crowded by cosolute molecules in a desiccated glassy matrix inspires several mechanisms, as described next and in Table 3.

**Table 3:** Examples of Mechanisms for Protecting Proteins in Glasses and Gels

**Protein**	**Protectant(s)**	**Drying**	**Method(s)**	**Mechanism/notes**	**Refs**
plasma	trehalose, glucose, sucrose	freeze-drying	DSC, FTIR, LS, NMR, OD	NS	(331)
MET16	trehalose, glucose	evaporation	CD, SRCD	NS	(231)
GB1	trehalose, glucose, sucrose, maltose, fucose, rhamnose, l-galactose, sorbitol, hexanediol	freeze-drying	DSC, NMR, TGA	water replacement	(238)
lysozyme	trehalose, sucrose, dextran	spray drying, lyophilization	DSC, FTIR, SAXS, WAXS	water replacement and anchorage	(105,106)
lysozyme	trehalose	freeze-drying	FTIR, Raman	water entrapment	(66,238,332−335)
GB1 and CI2	CAHS proteins	freeze-drying	DSC, NMR, TGA	volume restriction and electrostatic interactions	
lactate dehydrogenase and lipoprotein lipase	CAHS, globular proteins, Ficoll, and trehalose	evaporation, lyophilization	enzyme activity	NS	(67)
myoglobin	trehalose	evaporation	DSC, absorption	water entrapment	(312,326)
lysozyme, α-lactalbumin, metmyoglobin, RNase A	sol–gel matrix	-	CD, DSC	volume restriction, water entrapment	(102)
α_3_*W*, ubiquitin, cytochrome *c*	reverse micelle	-	NMR	volume restriction	(96)
frataxins, titin domain I27	polyacrylamide gel	-	fluorescence	volume restriction	(336)
H^+^-ATPase	trehalose	-	enzyme activity assay, viscometry	increased viscosity	(337)
myoglobin	trehalose	evaporation	absorbance	increased viscosity	(338)
myoglobin	trehalose	evaporation	FTIR	anchorage	(339)
lysozyme	glycerol, trehalose	freeze-drying	Raman, neutron scattering	anchorage	
lysozyme	trehalose, lactose, myoinositol	lyophilization	FTIR	water replacement	(340)
alkaline phosphatase	inulin, trehalose	spray drying	DSC, DVS	water replacement	(341)
lysozyme	sol–gel matrix	-	DSC, TGA-DTA-MS, CD	volume restriction	(342)

### 4.2 Water Retention

**Figure 8:**
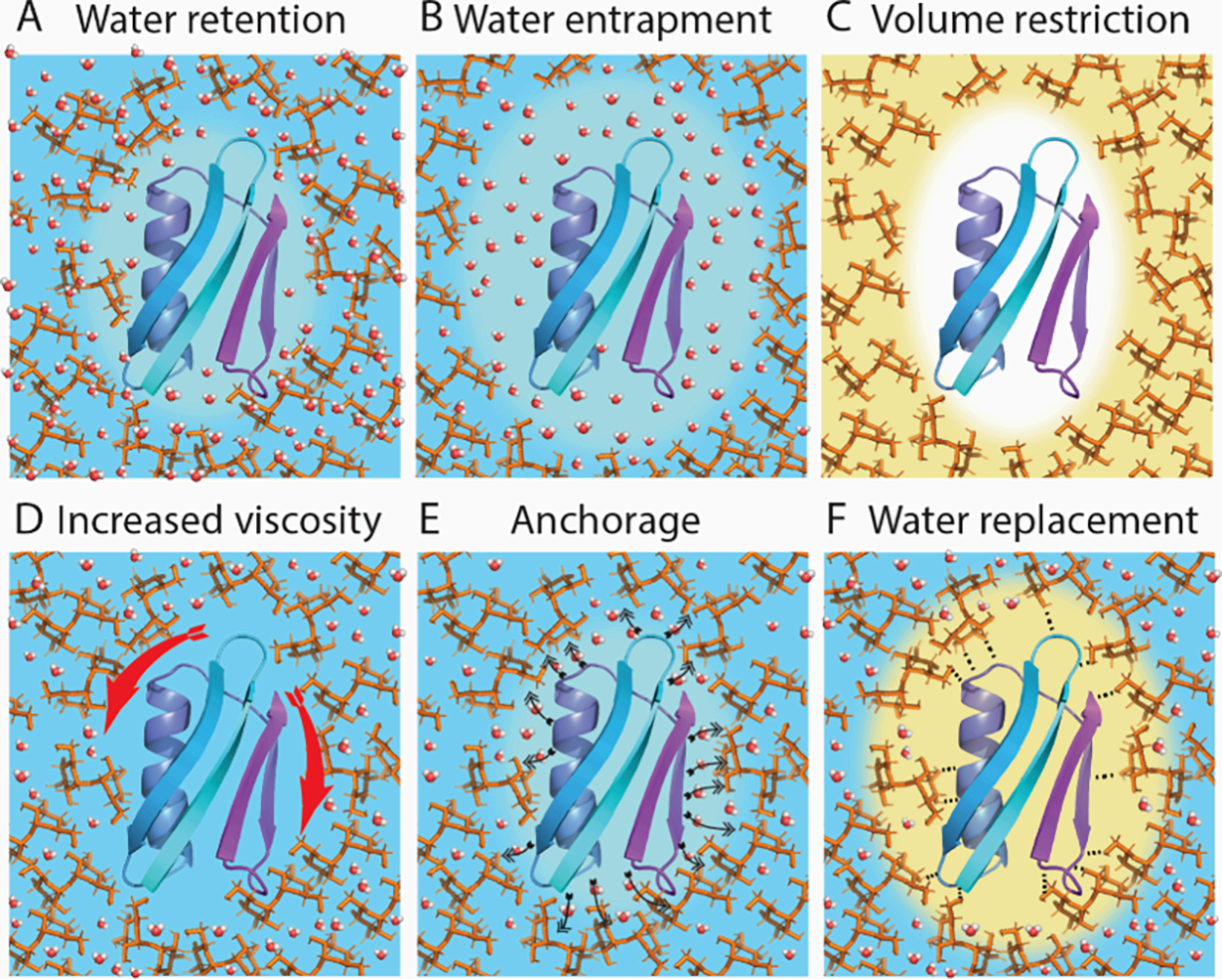
Schematics of hypotheses for protein stabilization by glassy matrices. (A) Water molecules are retained by the molecular glass, leading to stabilizing hydration of the protein. Water molecules are present both within the bulk glass and in proximity of the protein. (B) Water molecules are trapped near the protein interface by a glass that is dry compared with the protein surroundings. Entrapped water is strongly included in the protein environment, while the glass is dehydrated. (C) Steric exclusion restricts the protein to a smaller space (shown in white), supporting the stability of its compact native state. (D) The high viscosity in glass slows protein unfolding. Red arrows represent friction forces. (E) Water molecules anchor the protein to the glass matrix (illustrated by small arrows), restricting unfolding. (F) Water–protein hydrogen bonds are replaced by protectant–protein hydrogen bonds (shown by dashed lines), stabilizing the native state. Hypotheses are detailed in Sections 4.2 to 4.7.

One way to extend the ideas from stabilization by excluded crowders in solution to the glassy state is to consider a crowder’s ability to hold on to water upon desiccation. In the water retention hypothesis, a layer of water is maintained by protectant molecules (Figure 8). This residual water interacts with the target protein surface, preserving its stability.^324^ This mechanism requires that such protectants adsorb large amounts of water and therefore be more hygroscopic than nonstabilizing cosolutes and proteins. However, this hypothesis has tested negative for protein-based protectants from desiccation tolerant organisms because these protector proteins retain no more water than typical globular proteins.^324,325^ This mechanism is also unlikely for molecular glasses since their stabilizing efficacy usually, but not always,^326^ increases as water content decreases.^327−329^ Furthermore, glass-embedded proteins and lipid membranes likely retain equivalent amount of water as dry macromolecules on their own.^238,330^ In contrast to most other theories that explain protein stabilization within glass matrices (Sections 4.3 to 4.7), water retention diverges from the theoretical models of glass outlined in section 2.2. It does not invoke the impact of viscosity, reduced free volume, or glass cooperativity on protein stability.

### 4.3 Water Entrapment

A variation on water retention, water entrapment involves water trapped by the glass matrix as the protein “fights” with the matrix for water, as opposed to the available water being retained by the protectant molecules. The protein thus acts like a sponge that sequesters water from the glassy matrix, resulting in protein hydration (Figure 8).^326,343^ This idea resonates with the notion of CRRs introduced by Adam and Gibbs (Section 2.2), so that the protein possibly inhabits the higher mobility regions that engulf the more rigid CRRs. These more liquid-like regions may be more hydrated because of the inclusion of water molecules around the protein. Thus, the potential stabilizing effect of protein crowding (i.e., stabilization in dense liquid solutions by cosolute exclusion and preferential hydration) directly extends to vitrified glasses.^231,332,344^

The concept of water entrapment was introduced by Belton and Gil^332^ in the context of lysozyme’s interactions with a marginally hydrated trehalose glass, where the interaction was probed using FTIR and Raman spectrophotometry. Subsequent evidence from experiments utilizing calorimetry and viscometry reveal that embedded myoglobin draws in water from the adjacent glassy matrix.^326^ Additionally, molecular dynamic simulations of glass showcase how water molecules become ensnared between the primarily sugar-rich layer and the protein.^345−347^ Notably, protein–sugar interactions within this context are limited^312,344,346,347^ and weaker compared to stronger protein–water hydrogen bonds.^345^

### 4.4 Volume Restriction

In this model, the reduction in water content during vitrification and the concomitant accumulation of larger protectant molecules results in additional steric constraints. The augmented excluded volume interactions arising from higher protectant concentration reduce the available space for proteins in addition to reducing protectant free volume, as described by Doolittle’s model, eq 7. Confining a protein to a smaller volume stabilizes its compact native state.^348,349^ The stabilization results from the reduced conformational entropy of the denatured states, which increases their free energy with respect to the less affected native state.^349,350^

The influence of glass confinement on protein structural stability was demonstrated by Eggers and Valentine, who found that the thermal stability of proteins generally increases when encapsulated in a silica glass matrix.^102^ Since then, various experimental,^96,312,336,351−356^ theoretical,^349,350,357^ and computational^358,359^ efforts show that confinement enhances protein stability. However, as discussed (Section 3.2), the conditions used to remove the protein from confinement and competing interactions within the confined space can also lead to destabilization.

The increase in protein stability due to volume restriction coincides with an acceleration of folding rates,^350^ driven by the restricted search for the folded, native state in configurational space.^358^ However, when the cavity size becomes too small, folding slows due to the strong entrapment of the protein, preventing the necessary partial unfolding required to correct misfolded states.^350^ Consequently, to maintain protein stability, theoretical considerations require that the dimensions of the cavities within the glassy matrix that hold the protein must not fall below some critical size, beyond which folding is compromised.

### 4.5 Increased Viscosity

Conformational changes are required for protein unfolding. Protection by vitrification is often assumed to be the result of the large increase in viscosity below *T*_*g*_ (Figure 4A). As water content decreases, the viscosity of the mixture increases (in concert with *T*_*g*_ that also increases upon dehydration as described by the Fox equation, eq 1, and Gordon–Taylor equation, eq 2, Section 2.1), impeding movements large enough to trigger unfolding.^360^ In this purely kinetic mechanism, the increased viscosity causes greater friction between the protein and its environment,^337^ reducing diffusion.^338^ Viscosity depends on temperature as described by Andrade’s equation, eq 4, or the VFT models, eq 5. Slower diffusion also means that encounters between proteins become less frequent, diminishing the chance of their aggregation.

The rate constant for a reaction that relies only on diffusion, such as folding, decreases with viscosity according to Kramer’s theory, *k* = η^–1^*e*^Δ*E*^‡^/(*RT*)^ ∼ *e*^Δ*G*^‡^/(*RT*)^, where η is viscosity, Δ*E*^‡^ is the activation energy, and Δ*G*^‡^ is the activation free energy.^361,362^ The link to activation free energy is correct only if the viscosity follows the Arrhenius relation, η = η_∞_*e*^*E*^†^/(*RT*)^ ∼ *e*^*G*^†^/(*RT*)^, where η_∞_ is a pre-exponential constant and *E*^†^ and *G*^†^ are the viscosity activation energy and free energy, respectively. Given that the viscosity of the fragile glass formers (e.g., sugars) becomes exceedingly high below *T*_*g*_,^120,136,363^ it is tempting to conclude that friction between the protein and surrounding glassy matrix plays a substantial role in protein stabilization.

**Figure 9:**
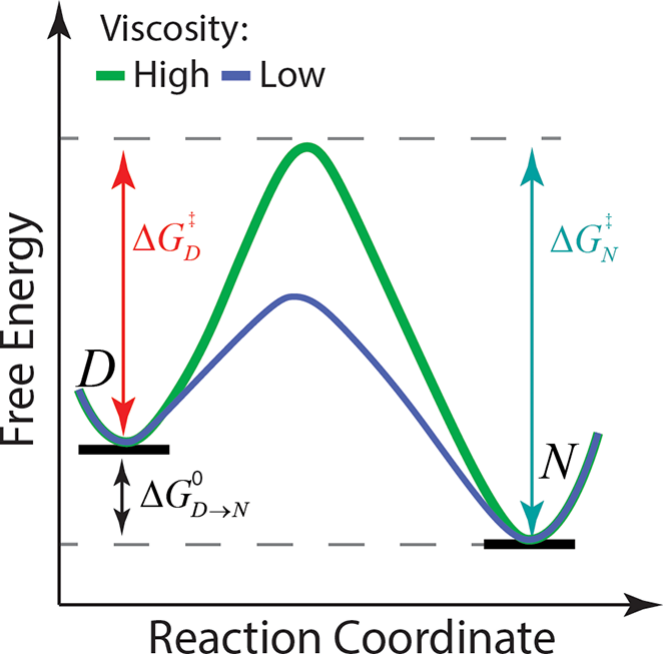
Schematic energy landscape for protein folding in media of high and low viscosity. In this example the denatured state, *D*, is unstable compared to the native state, *N*, by Δ*G*_*D* → *N*_^0^, which is in principle unaltered by viscosity, unlike the folding and unfolding activation energies (Section 4.5).

The relationship between protection and increased viscosity is satisfying, easy to grasp, and has been demonstrated for several proteins in crowded solutions,^360,362,364−366^ yet the presumed link between low water content and a high melting temperature in a protein-protectant mixture does not always hold.^326^ Even in cases where this relationship holds, it is not clear that viscosity alone is responsible for the increased stabilization. The fundamental objection is that at thermal equilibrium, higher viscosity (at least in its most simple definition) should slow both the folding and unfolding rates to the same extent, so that the equilibrium constant for folding is independent of viscosity, *K*_*eq*, *D*→*N*_ = *k*_*D*→*N*_/*k*_*N*→*D*_ = *e*^Δ*G*_*D*→*N*_^0^/(*RT*)^ (where Δ*G*_*D*→*N*_^0^ = Δ*G*_*N*_^‡^ – Δ*G*_*D*_^‡^), and protein stability (in terms of free energy) is unchanged (Figure 9). Of course, cosolutes often do change stability, but that is because they affect the free energy of the folded state, unfolded state, or both.^282^

### 4.6 Anchorage

The anchorage hypothesis combines elements from the entrapment and viscosity hypotheses. Here, protein motion becomes entwined with the glass matrix, or at least with adjacent CRRs (Section 2.2), through water molecules trapped between the protein and protectant (Figure 8).^344,345^ This coupling effectively “slaves” protein motion to that of the cooperative glassy matrix, linking the structural fluctuations associated with α-relaxation processes (Section 2.1) to the dynamics of the protein, up to 300 K for the CO molecule bound to carboxymyoglobin in trehalose glass^339^ and even 350 K for lysozyme in a glass composed of trehalose and glycerol.^367^ Although the correlation between protein stability and α-relaxation for a specific glass former at different compositions has been demonstrated, the degree of correlation can differ among various glass formers.^368,369^ By contrast, the relationship between the rate of protein unfolding and β-relaxation appears to be more consistent.^370^ This observation implies that protein stability in molecular glass is more closely associated with the local fluctuations characteristic of β-relaxation than the large-scale structural alterations of α-relaxation.

As already noted, reducing water content increases both the size of CRRs (as in the AG and Ginzburg’s models, Section 2.2) and glass viscosity (as in Andrade’s and VFT models), slowing both matrix- and protein dynamics.^371^ Adding small quantities of water or an alternative molecular plasticizer—substances that reduce the glass viscosity (e.g., glycerol)—to the matrix can also enhance protein stability by reinforcing the rigidity of the matrix, facilitated by robust hydrogen bonds between the glass-forming agent and the plasticizer.^21,345,372,373^

Within the framework of anchorage, differences in the efficacy of sugars in stabilizing proteins in the glassy state can be attributed to either increased matrix rigidity or increased protein-glass coupling. The rigidity idea suggests that the efficacy of fragile glass-forming materials surpasses that of stronger glass formers below *T*_*g*_ because fragile liquids experience a steeper increase in viscosity with decreasing temperature (Figure 4A).^367,374−376^ By contrast, it has been suggested that more fragile glass formers allow faster conformational changes with higher probability, which is detrimental to protein stability.^367^ The coupling notion implies that the number of water molecules shared by the protein and glass depends on the sugar and that stronger coupling stabilizes the protein. Nonetheless, both considerations explain the discrepancies in sugar stability. For instance, trehalose is more fragile than many other disacharides^376^ but also shares more water molecules than sucrose,^346^ likely due to the higher affinity of trehalose for water.^377^ This duality makes it challenging to determine which property leads to trehalose’s efficacy compared to other sugars. The inconsistent relationships between glass composition, including hydration level, sugar, and polyol content and the extent of glass-induced stability increase highlight some of the unresolved questions concerning glasses and dry protein stability.^247^

### 4.7 Water Replacement

Water replacement is the only hypothesis that emphasizes direct protein–protectant interactions. Specifically, it posits that protectants function by substituting water–protein hydrogen bonds with protectant-protein hydrogen bonds upon desiccation.^120,340,378,379^ This hypothesis goes beyond current glass theories, which focus on properties of the glass former, by emphasizing the coupling between protein and protectant through direct molecular interactions.

The idea was proposed by Crowe et al.,^380−382^ who demonstrated that stabilization of lipid membranes by sugars results from the establishment of hydrogen bonds between sugar hydroxyls and the phospholipid head groups of lipids.^118,330,382−391^ The notion of stabilization via direct hydrogen bonding was expanded to encompass proteins.^119^ For instance, changes in the amide II band of lysozyme upon dehydration are mitigated in the presence of trehalose.^340^ Further evidence comes from the NMR-based observation that degree of protection is related to the number of hydroxyl groups in the protectant.^238^ Likewise, CD data reveal that the glass matrix influences protein stability through intermolecular chemical interactions.^231^

The proposed direct interactions are often invoked to rationalize variation in protein stabilization observed among molecular glasses with similar *T*_*g*_ values.^341^ Nevertheless, it remains challenging to dismiss the influence of the protectant’s fragility (and consequently, viscosity and relaxation dynamics),^374,376^ as well as potential contributions from the other hypotheses.

## 5 EMBEDDED IN GLASS — PROTEIN PERSPECTIVE

### 5.1 Alternate Protein Structures

The solvating environment plays a critical role not only in protein stability, shifting the equilibrium toward or away from the native state, but also in the structure of the compact native state and, even more so, the structures in the extended denatured ensemble. By analogy to compaction of intrinsically disordered proteins under crowded conditions,^322^ the denatured state confined within a glassy matrix experiences a substantial reduction in conformational entropy (Section 4.4), which results in compaction.^348,350^ Interestingly, simulations of a protein with a β-hairpin native fold, derived from the C-terminal of protein G, suggest that volume restriction on its own is sufficient to impact the denatured state by favoring structural elements in the ensemble with native-like structure.^358^

**Figure 10:**
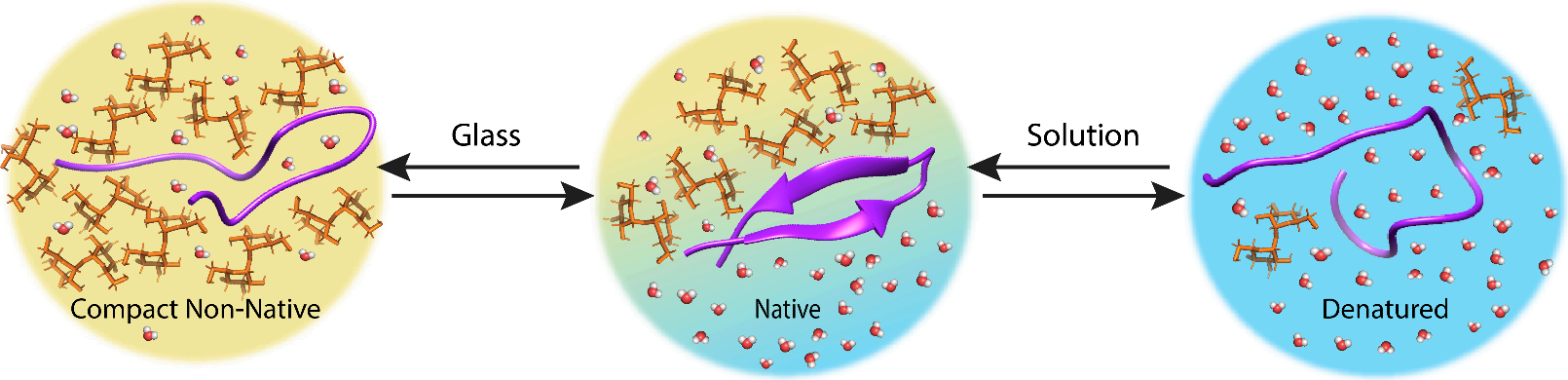
Scheme of the β-hairpin model protein MET16 solvated in aqueous solution and embedded in a sugar glass. The native state (center) is conserved between solution and glass environments. In solution, the denatured state is extended (right), while within the glass matrix, it adopts a more compact structure (left) that more closely resembles the native state structure.

Confirmation of the retention of native-like structure in the denatured state is provided by circular dichroism (CD) data on another β-hairpin model protein, MET16, embedded in trehalose and glucose glasses.^231^ These experiments reveal that the denatured state in the glass matrix closely resembles the native state and differs from the denatured state in aqueous solution (Figure 10). Similarly, novel partially unfolded trapped states were spectroscopically observed for myoglobin in sol–gels.^392^ This type of compaction may reduce the amount of harmful, aggregation-prone misfolded conformations (Section 5.2). In addition, compaction of the denatured state may reduce the folding time because the conformational search is more confined.

Finally, the compaction of MET16’s denatured state within the glassy matrix changes the folding mechanism, from predominantly entropically driven in solution to enthalpy driven in the glass. This mechanistic change reverses the temperature dependence of the folding free energy and leads to increased protein stabilization in the glass at ambient temperatures.^231^

Although the compact denatured states of MET16 in trehalose and glucose glass are indistinguishable, trehalose affords more stabilization, which is strongly enthalpy driven. This sugar-specific shift in equilibrium thermodynamics suggests that the roles played in solution by hydrophobic contacts and water release upon folding are replaced in the glass by specific interactions between the glass and the restricted folded and denatured states. The differences between sugars in the extent of stabilization suggest that the matrix is not simply inert. Instead, nonsteric intermolecular glass–protein interactions play a role in protein stabilization.

Secondary structure content from FTIR data on dry samples shows substantial structural differences between aqueous solutions and sugar glass for larger globular proteins, including bovine serum albumin, myoglobin, and lysozyme.^393−396^ When these proteins are combined with sugar and polyol glass formers like sucrose, trehalose, maltose, and glycerol in their desiccated form, a marked increase in α-helix content is observed compared to the same proteins desiccated in the absence of protectant. Furthermore, changes in the content of α-helix, β-sheet, and various types of unordered structures are noted between aqueous solutions and sugar glass.^393^ These structural changes also impact the temperature dependence of folding, generally extending the stability of α-helix and β-sheet structures to higher temperatures.

### 5.2 Intra-Protein Interactions

Beyond modifying protein structure and interactions with their environment, protein confinement by dense and crowded solutions, as well as integration into a protective glass matrix, also impact interactions between protein residues within the protein and among neighboring proteins. As a result, the amorphous glass matrix modulates protein stability and protects against unfolding and irreversible aggregation, processes that are usually detrimental to function.

**Figure 11:**
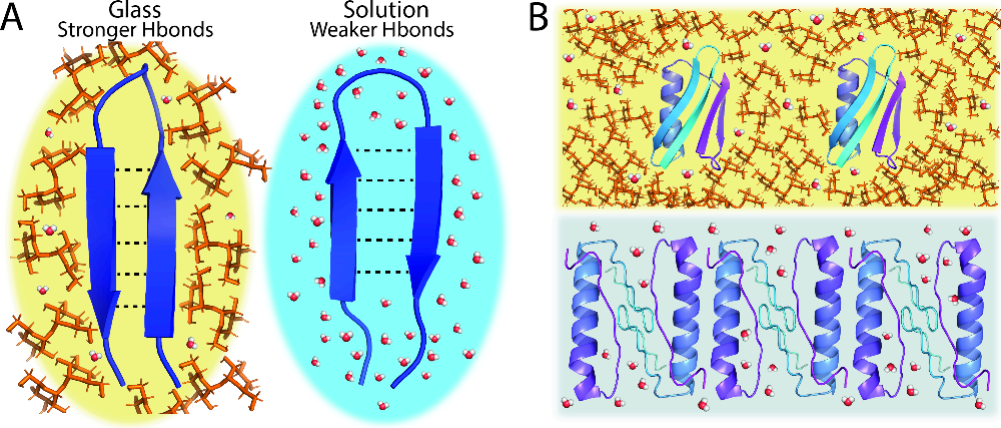
Schematics of the impact of molecular glass on protein–protein interactions. (A) Compaction by a sugar glass leads to stronger intra-protein hydrogen bonds in the matrix (left panel, dashed lines), whereas hydrogen bonds are weaker in aqueous solution (right). (B) Protein dilution by glassy protectant (top) versus aggregation of dry protein in the absence of protectants (bottom).

The increase in number and strength of intra-protein interactions by protectants is evident in simulations of highly crowded solutions, even at concentrations below the glass transition. A study combining MD simulation and NMR demonstrates that polyols and sugars reduce the length, and hence increase the strength, of intra-protein hydrogen bonds (Figure 11A).^397^ In another simulation study, an osmotic pressure of 3.9 Osmolal exerted by the sugar alcohol sorbitol was shown to considerably strengthen intra-protein hydrogen bonds, leading to stabilization.^398^ Notably, this increase in hydrogen bonding strength seems to have a more pronounced impact on intrabackbone bonds compared to those involving side chains.^397,398^

Furthermore, by disproportionately weakening the protein’s hydrogen bonds in the native and denatured states with the surrounding solution, polyols and sugars can effectively mitigate the loss of hydrogen bonds between the protein and its environment upon folding.^241^ The implication is that even in an environment that affords the protein fewer and weaker hydrogen bonds (as is typically the case for glass formers), native state stability can be enhanced simply by destabilizing the unfolded state more than the folded state.

The protection provided by sugar glass against unfolding is particularly notable for residues engaged in internal protein hydrogen bonds, especially residues fostering two or more hydrogen bonds.^238^ DSC and TGA, together with liquid-observed vapor exchange NMR spectroscopy reveal that protection afforded to residues by the sugar glass is linked to the number of intramolecular hydrogen bonds within the protein.^238^ This protection implies an increase in native state stability within the glass, driven by the reinforcement of intra-protein hydrogen bonds, compared to the liquid state. This strengthening of intra-protein hydrogen bonds appears to be a consistent trend among sugar glass formers.

### 5.3 Inter-Protein Interactions

In dilute solution, proteins tend to aggregate when interactions between neighboring proteins become more favorable than their interactions with the solvent.^399^ Most cells maintain an extremely concentrated environment at their normal hydration levels,^27,400^ causing proteins to be practically supersaturated.^401^ Even before desiccation, a small decrease in water content often leads to aggregation. Under extreme desiccation, where most of the water is removed, proteins are left with a minimal hydration layer that barely separates one protein from another. Consequently, desiccation can exacerbate aggregation by bringing proteins into close proximity, increasing intermolecular interactions and the likelihood of sticking to each other.

Molecular glass matrices modulate the inter-protein interactions that lead to aggregation at elevated protein concentrations. Molecular glasses have been shown to mitigate aggregation of a diverse range of proteins.^51,402−406^ A factor contributing to this protection is the physical separation facilitated by the matrix (Figure 11B).^399^ In essence, diluting the protein with the protectant maintains some distance between proteins, thus protecting them from aggregation.^407^ For instance, simulations demonstrate that trehalose in its glassy state weakens inter-protein hydrogen bonds, thereby limiting protein–protein contacts.^345,408^

In molecular glasses, protection from aggregation relies not only on protein dilution but also on enhancement of protein stability. Because inter-protein interactions often involve nonspecific hydrophobic forces,^40^ these short-range interactions contribute and can even dominate aggregation at high protein concentrations.^409^ Conversely, aggregation is mitigated by additives that exhibit limited binding to the protein interface, because they promote protein compaction and thus reduce interactions between exposed hydrophobic protein moieties.^410^ Specific interactions, such as electrostatic forces and hydrogen bonds, may also contribute to protein aggregation, but their impact is less predictable because they strongly depends on the specific protein.^238,399,411^

Aggregation, including the formation of amyloids, generally proceeds from partially unfolded protein states.^412,413^ Consequently, increasing the stability of the native state, which reduces the frequency of unfolding events, also alleviates aggregation.^240,414^ Notably, during desiccation, including in processes such as lyophilization, unfolding can lead to aggregation, underscoring the advantage of inhibiting conformational changes.^406^ Inhibition of conformational changes can be achieved by adding sugars that stabilize proteins.^238^ For instance, SAXS measurements of lysozyme dried in sucrose suggest the preservation of its native state.^106^ In contrast, lysozyme dried in the absence of sugar displays significant structural distortions compared to the native state in solution.^415^ This observation suggests that, indeed, diluting the protein with sugars in the glassy state achieves both protection from aggregation and increased protein stability by mitigating destabilizing protein–protein interactions.

## 6 CONCLUSIONS

Desiccation protection is essential for the survival of organisms experiencing harsh conditions and imperative in technological applications in the food, pharma, and biomedical industries. Organisms have evolved various strategies to protect their macromolecules from the detrimental effect of drying. One of the most common invokes the formation of amorphous glassy matrices made from molecularly small metabolites (most notably sugars) alone or in combination with intrinsically disordered proteins (e.g., LEA and CAHS proteins). The idea that nonvolatile additives that form amorphous vitrified media can overcome the detrimental impacts of drying is shown by their implementation in industry. A host of molecular glassy matrices, silica glass, and aerogels have been suggested as protective media. Due to their simplicity of application, the use of glassy and amorphous media of nonvolatile solutes has become an attractive solution, and research in this area is poised to result in the discovery of robust, hypothesis-driven strategies of protection.

Despite advances in developing glasses for desiccation protection, the molecular mechanisms of protection remain controversial and comprise an active area of research. Many of the mechanisms described in this review highlight the difficulty in pinpointing their details. The extent of involvement of the matrix and residual water as well as the details of the “fight over water” between protein and matrix elements are difficult to deconvolute. One problem is the separation of time and length scales. In solution, proteins fold much more slowly than the solvent relaxes around them, whereas in glasses, relaxation slows, and proteins may even seem immobile, yet experiments show that ensembles of protein configurations correspond, at least in part, to native and denatured states.

Another persistent question is the quality and degree of protection and preservation that glasses afford. It has been known for years that trehalose has been selected as a protectant by many organisms, yet what makes this sugar superior to others is not completely understood. More generally, it is unclear which glass properties most closely correspond to the protection efficacy. It is tempting to choose easily measured properties, such as the glass transition temperature or molecular inertness, but unfortunately these properties do not always correspond to the most protective glass formers.

Beyond understanding how the glass impacts the embedded proteins, a relevant aspect that has not been widely explored is the way that proteins modify the properties of the matrix. This facet is important because in the vitrified state, the ratio of protein to glass-forming molecules is large, suggesting that almost every glass former is in close contact with a protein. It is hard to imagine how the glass properties would not then be impacted by the protein. Can these effects teach us about the properties that make a particularly good protectant?

Another important aspect that is only beginning to be explored is the advantages afforded by mixtures of glass formers. Specifically, mixtures of sugars and plasticizers such as glycerol are known to improve the protective efficacy of the glass, but the mechanism is unresolved. It is also known that some protective proteins form gels that may act in combination with other additives such as trehalose. Could the cohabitation of gel forming proteins and molecularly small glass formers lead to better protective properties?

The glassy matrix is complex and poses many challenges, both theoretical and experimental, yet the importance of its protective propensity indicates that a better molecular-level understanding of how the matrix stabilizes proteins will lead to a better grasp of biology and thus pave the way to the development of improved glasses with an even wider range of applications.

## LIST OF ABBREVIATIONS

AG: Adam and Gibbs (model)
CAHS: cytosolic abundant heat soluble
CD: circular dichroism (spectroscopy)
CRR: cooperatively rearranging region
GD: Gibbs and DiMarzio (model)
DSC: differential scanning calorimetry
DVS: dynamic vapor sorption
FTIR: Fourier-transform infrared (spectroscopy)
GB1: B1 domain of streptococcal protein G
GBP: *Escherichia coli* glucose binding protein
LEA: late embryogenesis abundant
LS: light scattering
MD: molecular dynamics
NMR: nuclear magnetic resonance (spectroscopy)
NS: not stated
OD: oxidative damage
PEG: polyethylene glycol
SAXS: small-angle X-ray scattering
SRCD: synchrotron radiation CD
TEOS: tetraethoxysilane
TGA: thermogravimetric analysis
TMOS: tetramethoxysilane
TNM: Tool–Narayanaswamy–Moynihan (model)
VFT: Volgel–Flucher–Tammann (model)
WAXS: wide-angle X-ray scattering
WLF: Williams–Landel–Ferry (model)
